# Safety of Invasive Procedures During Adult Extracorporeal Membrane Oxygenation: A Systematic Review

**DOI:** 10.3390/jcm15124792

**Published:** 2026-06-20

**Authors:** Giuseppe Neri, Giuseppe Mazza, Helenia Mastrangelo, Jessica Ielapi, Federico Longhini, Vincenzo Bosco, Alessandro Russo, Francesca Serapide, Isabella Aquila, Matteo Antonio Sacco, Zaninni Caroleo, Andrea Bruni, Eugenio Garofalo

**Affiliations:** 1 Department of Medical and Surgical Sciences, “Magna Graecia” University of Catanzaro, 88100 Catanzaro, Italy; giuseppeneri91@gmail.com (G.N.); giuseppe.mazza@unicz.it (G.M.); jessica.ielapi22@gmail.com (J.I.); flonghini@unicz.it (F.L.); vincenzo.bosco@unicz.it (V.B.); a.russo@unicz.it (A.R.); f.serapide@unicz.it (F.S.); isabella.aquila@unicz.it (I.A.); matteosacco@unicz.it (M.A.S.); 2 ASP Catanzaro, 88100 Catanzaro, Italy; heleniamastrangelo@gmail.com; 3 Department of Pharmacy and Health and Nutrition Sciences, University of Calabria, 87036 Cosenza, Italy; caroleozaninni@gmail.com (Z.C.); andrea.bruni@unical.it (A.B.)

**Keywords:** ECMO, extracorporeal membrane oxygenation, extracorporeal life support, invasive procedures, bleeding, transfusion, thrombosis, thoracic surgery, abdominal surgery, airway procedures, lung transplantation

## Abstract

**Background/Objectives:** Adult patients supported with extracorporeal membrane oxygenation (ECMO) frequently require invasive diagnostic, therapeutic, surgical, or bedside procedures during ongoing extracorporeal support. These procedures are clinically challenging because ECMO-related anticoagulation, platelet dysfunction, acquired coagulopathy, and circuit-related coagulation activation may increase both bleeding and thrombotic risks. This systematic review evaluated the safety of invasive procedures performed during adult ECMO support, excluding tracheostomy/tracheotomy because this procedure has recently been addressed in a dedicated systematic review. **Methods:** A systematic search of PubMed/MEDLINE and Scopus was performed. The final bibliographic data collection was completed in April 2026. Studies were eligible if they included adult ECMO or extracorporeal life support patients undergoing invasive procedures during ongoing ECMO support, or with ECMO used as procedural support, and reported at least one procedure-specific safety outcome. Primary outcomes were procedure-related complications, bleeding, major bleeding, and transfusion requirements. Secondary outcomes included thrombotic and circuit-related complications, oxygenator exchange, reintervention, reoperation, procedural failure, ECMO duration, intensive care unit and hospital length of stay, and mortality. **Results:** The final qualitative synthesis included 46 studies, comprising 26 studies from PubMed/MEDLINE and 20 additional unique studies from Scopus. Included procedures were grouped into six domains: airway, bronchoscopic, and tracheobronchial procedures; thoracic surgery and lung resections; abdominal surgery, gastrointestinal endoscopy, and decompressive laparotomy; lung transplantation and perioperative extracorporeal life support; cardiovascular, vascular, pulmonary embolism-related, and mechanical circulatory support-related procedures; and mixed non-cardiac surgery. Airway and bronchoscopic procedures generally showed high procedural success in selected cohorts, although registry-level tracheal procedure data reported hemorrhagic complications in 26.0% and surgical-site bleeding in 13.0%. Emergency thoracic and abdominal procedures carried the highest bleeding, transfusion, reintervention, and mortality burden. Lung transplantation studies showed that ECMO can be integrated into perioperative pathways, but hemothorax, transfusion, thromboembolism, and anticoagulation strategy remained central safety issues. **Conclusions:** Invasive procedures during adult ECMO are feasible in selected patients and experienced centers, but procedural safety varies markedly by procedure type, urgency, baseline disease severity, and anticoagulation strategy. A procedure-centered, multidisciplinary approach with individualized anticoagulation management and careful planning is essential.

## 1. Introduction

Extracorporeal membrane oxygenation (ECMO) is increasingly used in adult critical care as temporary support for severe respiratory and/or circulatory failure. Improvements in ECMO technology, patient selection, anticoagulation strategies, and multidisciplinary expertise have progressively expanded its application beyond conventional rescue indications. ECMO is now used not only in refractory respiratory or cardiac failure, but also as perioperative support for complex non-cardiac surgery, high-risk airway interventions, thoracic surgery, abdominal emergencies, lung transplantation, selected cardiovascular procedures, and mechanical circulatory support-related interventions [1,2,3,4,5,6,7,8,9].

As the number and complexity of adult ECMO-supported patients increase, invasive diagnostic, therapeutic, surgical, and bedside procedures are frequently required during ongoing extracorporeal support. These procedures may be necessary for diagnosis, source control, bleeding control, airway patency, tissue sampling, decompression of abdominal compartment syndrome, transplantation, or treatment of disease-related and ECMO-related complications [3,4,5,6,7,8,10,11,12,13,14,15,16,17,18]. In clinical practice, the relevant question is therefore not only whether ECMO patients are generally at risk of bleeding or thrombosis, but whether a specific invasive procedure can be safely performed during ECMO, under which procedural and anticoagulation conditions, and with what expected complication profile.

The procedural management of ECMO-supported patients is challenging because ECMO is associated with acquired and dynamic hemostatic alterations. These include exposure to systemic anticoagulation, thrombocytopenia, platelet dysfunction, acquired von Willebrand syndrome, hemodilution, inflammation, and circuit-related activation of coagulation and fibrinolysis. At a mechanistic level, blood exposure to artificial surfaces and non-physiological shear stress may promote contact-pathway and platelet activation while also causing shear-induced loss of high-molecular-weight von Willebrand factor multimers. Concomitant platelet consumption or functional exhaustion, fibrinolytic dysregulation, and endothelial or glycocalyx injury related to shock and extracorporeal circulation may further amplify both hemorrhagic and thrombotic vulnerability. These mechanisms coexist with the underlying critical illness and may increase the risk of peri-procedural bleeding, transfusion requirement, hemothorax, surgical-site bleeding, reintervention, reoperation, and mortality [10,11,19,20,21]. Conversely, reduction or interruption of anticoagulation around invasive procedures may expose patients to thrombotic complications, including deep-vein thrombosis, circuit thrombosis, oxygenator dysfunction, cannula-associated thrombosis, thromboembolic events, and stroke [11,12,19,22,23].

The available evidence is heterogeneous and distributed across different procedural domains. Airway and bronchoscopic studies suggest that ECMO may facilitate high-risk rigid bronchoscopy, bronchotracheal stenting, whole-lung lavage, transbronchial lung cryobiopsy, and complex airway surgery when conventional ventilation is unsafe or impossible [3,4,14,15,16,24,25]. Thoracic surgery studies describe the use of ECMO during non-elective thoracic surgery, lung abscess resection, emergency pulmonary lobectomy or pneumonectomy, lung volume reduction surgery, thoracic bleeding management, VATS, and major cardiopulmonary resections [5,6,10,11,17,21,22,26,27,28,29,30,31]. Abdominal studies report emergency laparotomy, abdominal exploration, decompressive laparotomy for abdominal compartment syndrome, open abdomen therapy, and endoscopic management of gastrointestinal bleeding during ECMO support [7,13,18,32,33,34,35]. Lung transplantation represents a distinct procedural field in which ECMO may be used intraoperatively or perioperatively, with bleeding, transfusion burden, hemothorax, thromboembolism, renal replacement therapy, and anticoagulation strategy remaining central safety issues [8,9,12,19,20,23,36]. Finally, cardiovascular, vascular, pulmonary embolism-related, and mechanical circulatory support procedures include durable left ventricular assist device implantation after extracorporeal life support, robot-assisted coronary artery bypass with peripheral ECMO, thoracoabdominal aortic replacement, and invasive treatment strategies for critical pulmonary embolism requiring ECMO [37,38,39,40,41,42,43].

Despite this growing body of literature, most studies are retrospective, single-center, procedure-specific, registry-based, or comparative observational cohorts. Outcome definitions are inconsistent. Bleeding may be reported as intraoperative blood loss, postoperative hemorrhage, hemothorax, surgical-site bleeding, major bleeding, transfusion requirement, re-exploration, or clinically relevant bleeding requiring endoscopic, radiologic, or surgical treatment. Similarly, thrombotic and circuit-related complications are variably reported. This heterogeneity limits quantitative pooling but supports the need for a systematic, procedure-centered qualitative synthesis.

Tracheostomy and tracheotomy during ECMO are important invasive procedures and have recently been addressed in a dedicated systematic review [44]. Therefore, the present systematic review does not aim to duplicate that evidence. Instead, it focuses on other invasive procedures performed during adult ECMO support, while including tracheal, airway, and bronchoscopic procedures when the index intervention was not tracheostomy or tracheotomy and procedure-specific safety outcomes were extractable.

The aim of this systematic review was to evaluate the safety of invasive procedures performed during adult ECMO support. The primary outcomes were procedure-related complications, procedure-related bleeding, major bleeding, and transfusion requirements. Secondary outcomes included anticoagulation management, thrombotic and circuit-related complications, oxygenator exchange, reintervention or reoperation, procedural failure, ECMO duration, intensive care unit and hospital length of stay, and mortality.

## 2. Materials and Methods

### 2.1. Study Design and Reporting

This systematic review was designed to evaluate the safety of invasive procedures performed during adult extracorporeal membrane oxygenation support. The review was conducted and reported in accordance with the Preferred Reporting Items for Systematic Reviews and Meta-Analyses 2020 statement [45]. The completed PRISMA 2020 checklist is provided as Supplementary File S2. The review focused on procedure-specific safety outcomes rather than on general ECMO-related bleeding, thrombosis, or anticoagulation management. The review protocol was not prospectively registered. Although protocol registration is not mandatory for conducting a systematic review, the absence of prospective registration limits the external verification of predefined eligibility criteria, outcome prioritization, and planned synthesis methods. To enhance methodological transparency, the present review was conducted according to PRISMA 2020 recommendations, and the complete eligibility criteria, search strategies, study selection process, data extraction framework, and planned qualitative synthesis are reported in the manuscript and Supplementary Materials.

### 2.2. PICO Framework and Research Question

The review question was structured according to the PICO framework. The population consisted of adult patients receiving ECMO or extracorporeal life support, including veno-venous ECMO, veno-arterial ECMO, veno-arterial–venous ECMO, or extracorporeal support used as peri-procedural support.

The intervention or exposure was any invasive diagnostic, therapeutic, surgical, bronchoscopic, bedside, cardiovascular, vascular, abdominal, thoracic, airway, transplantation-related, pulmonary embolism-related, or mechanical circulatory support-related procedure performed during ongoing ECMO support or with ECMO used as procedural support.

No mandatory comparator was required. When available, comparator groups included patients undergoing similar procedures without ECMO, patients treated with different procedural approaches, different ECMO configurations, different anticoagulation strategies, or ECMO-supported patients not undergoing the index procedure.

The primary outcomes were procedure-related complications, procedure-related bleeding, major bleeding, and transfusion requirements. Secondary outcomes included anticoagulation interruption or modification, thrombotic complications, circuit thrombosis, oxygenator exchange, cannula-associated thrombosis, stroke, reintervention, reoperation, procedural failure, ECMO duration, intensive care unit length of stay, hospital length of stay, and mortality.

The research question was: in adult patients receiving ECMO support, what is the reported safety profile of invasive procedures performed during ECMO?

### 2.3. Information Sources and Search Strategy

A systematic literature search was conducted in PubMed/MEDLINE and Scopus. The search strategy was developed to identify studies evaluating invasive procedures performed during adult ECMO support and reporting procedure-related safety outcomes. Search terms combined concepts related to ECMO/ECLS, invasive procedures, and safety outcomes, including bleeding, transfusion, thrombosis, circuit complications, oxygenator exchange, reintervention, reoperation, and procedural failure. Final bibliographic data collection and database searches were completed in April 2026. The complete PubMed/MEDLINE and Scopus search strategies, including all search terms, Boolean operators, field restrictions, applied limits, search dates, and numbers of retrieved records, are provided in Supplementary File S1.

### 2.4. Eligibility Criteria

Studies were considered eligible if they met all of the following criteria: adult patients receiving VV-ECMO, VA-ECMO, VAV-ECMO, ECLS, or extracorporeal support used as peri-procedural support; invasive procedures performed during ongoing ECMO support or with ECMO used as procedural support; reporting of at least one procedure-related safety outcome; and original clinical study design, including retrospective cohorts, prospective cohorts, registry analyses, case series, and comparative observational studies.

Eligible procedures included airway and bronchoscopic procedures, tracheobronchial interventions, transbronchial lung biopsy or cryobiopsy, thoracic surgery, lung resections, chest drainage or thoracic interventions, abdominal surgery, decompressive laparotomy, endoscopic management of gastrointestinal bleeding, lung transplantation with intraoperative or perioperative ECMO/ECLS, pulmonary embolism-related invasive treatment strategies, vascular or cardiovascular procedures, mechanical circulatory support-related procedures, and mixed non-cardiac surgical procedures.

Studies focusing on tracheostomy or tracheotomy during ECMO were excluded because this procedure has recently been addressed in a dedicated systematic review [44]. Studies including tracheal, bronchoscopic, or airway procedures were not excluded when the index procedure was not tracheostomy or tracheotomy and procedure-specific safety outcomes were extractable.

Studies were excluded if they involved pediatric or neonatal ECMO, ECMO cannulation or decannulation as the index procedure, anticoagulation-only strategies without a specific invasive procedure, general ECMO outcomes without procedure-specific data, post-cardiotomy ECMO without a defined procedure performed during support, case reports with a single patient, reviews, editorials, letters, protocols, and studies in which ECMO-specific procedural safety outcomes were not separately extractable.

### 2.5. Screening and Eligibility Assessment

**Figure 1:**
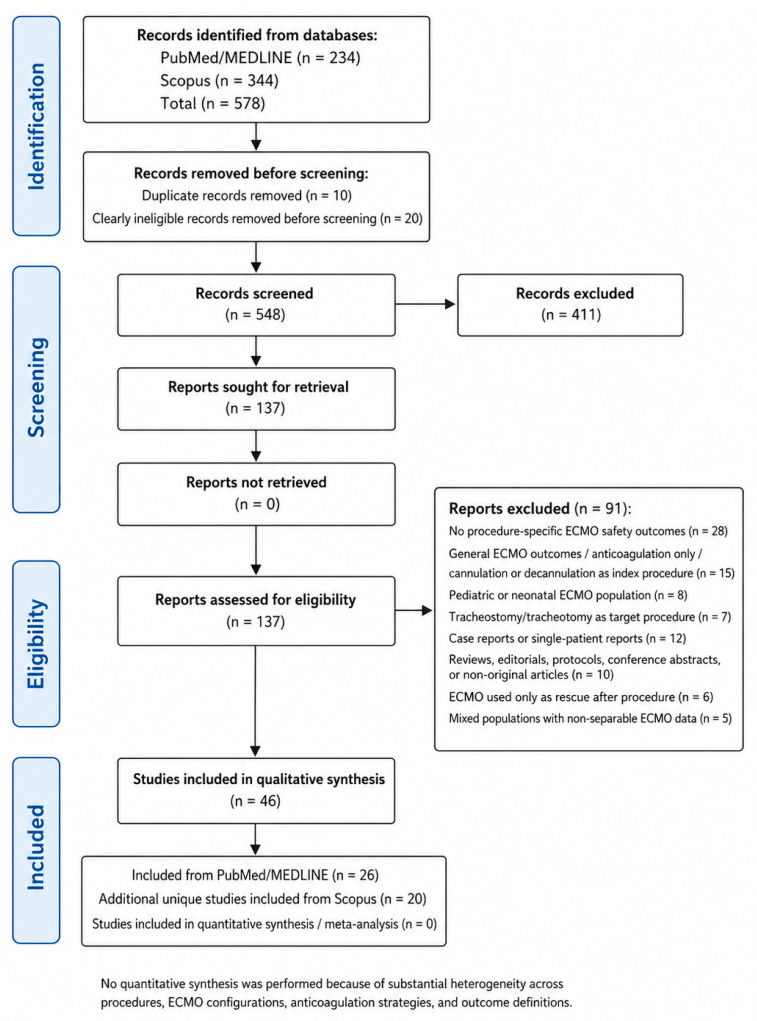
PRISMA 2020 flow diagram of study selection. The PubMed/MEDLINE search identified 234 records and the Scopus search identified 344 records, for a total of 578 records. After removal of 10 duplicates and 20 records excluded before screening because they were clearly outside the scope of the review, 548 records underwent title and abstract screening. Overall, 137 reports were assessed for eligibility. Ninety-one reports were excluded after full-text assessment, leaving 46 studies included in the final qualitative synthesis, comprising 26 studies from PubMed/MEDLINE and 20 additional unique studies from Scopus. No quantitative meta-analysis was performed because of substantial clinical and methodological heterogeneity.

Study selection was performed in sequential phases. After removal of records excluded before screening and duplicate records, titles and abstracts were independently screened by two reviewers (G.N. and G.M.) according to the predefined eligibility criteria. Records judged eligible or potentially eligible by at least one reviewer were retrieved for full-text assessment. Full-text articles were then independently assessed by the same two reviewers. Disagreements at any stage of the selection process were resolved by discussion and, when necessary, by consultation with a third senior reviewer (E.G.). Reasons for exclusion at the full-text stage were recorded and categorized according to PRISMA 2020 recommendations. The numerical results of the selection process are reported in Section 3.1 and Figure 1.

### 2.6. Data Extraction

Data extraction was performed independently by two reviewers (G.N. and G.M.) using a standardized data extraction form. Extracted data were cross-checked for accuracy and completeness by a third reviewer (E.G.). Any discrepancy was resolved by consensus among the reviewers. When required, additional consultation was obtained from senior authors with expertise in ECMO, intensive care, and perioperative medicine (F.L. and A.B.).

Data were extracted using a standardized data extraction form. The following information was collected from each study: first author and year of publication, country and study setting, study design, study period, sample size, adult ECMO population, ECMO configuration, indication for ECMO, type of invasive procedure, procedural setting, anticoagulation strategy when reported, bleeding complications, major bleeding, transfusion requirements, thrombotic complications, circuit complications or oxygenator exchange, procedural success or failure, reintervention or reoperation, ECMO duration, ICU length of stay, hospital length of stay, and mortality.

When studies included mixed populations, only ECMO-specific data were extracted whenever separable. For mixed cardiopulmonary bypass/ECMO studies, only ECMO-specific cases were considered when data were extractable. Studies in which ECMO-specific procedural outcomes were not separable were excluded from the synthesis.

Comparative statistics, including *p*-values, odds ratios, hazard ratios, confidence intervals, and survival analysis results, were extracted and reported descriptively when available.

### 2.7. Outcomes

The primary outcomes were overall procedure-related complications, procedure-related bleeding, major bleeding, and transfusion requirements.

Secondary outcomes included anticoagulation interruption or modification, thrombotic complications, circuit thrombosis, oxygenator exchange, cannula-associated thrombosis, stroke, reintervention, reoperation, procedural failure, ECMO duration, ICU length of stay, hospital length of stay, and mortality.

Because outcome definitions varied substantially across studies, bleeding and major bleeding were extracted according to the definitions used by the original authors. When a study explicitly used a formal classification system, such as ELSO, ISTH, BARC, or another author-specified definition, that definition was recorded as reported. No post hoc reclassification of bleeding events according to a single uniform definition was performed. When formal criteria were not provided, individual reported events—including hemothorax, surgical-site bleeding, postoperative hemorrhage, transfusion requirement, re-exploration or reintervention for bleeding, and clinically relevant bleeding requiring endoscopic, radiological, or surgical treatment—were extracted and retained as distinct procedure-specific safety outcomes whenever separately available.

### 2.8. Procedural Classification

Included studies were grouped into predefined procedural domains to provide a clinically practical synthesis for ICU, anesthesia, surgical, and ECMO teams. The procedural domains were: airway, bronchoscopic, and tracheobronchial procedures; thoracic surgery and lung resections; abdominal surgery, gastrointestinal endoscopy, and decompressive laparotomy; lung transplantation and perioperative ECLS; cardiovascular, vascular, pulmonary embolism-related, and mechanical circulatory support-related procedures; and mixed non-cardiac surgical procedures.

Studies involving more than one procedural type were classified according to the predominant procedure or, when appropriate, described across multiple relevant domains in the narrative synthesis.

### 2.9. Risk of Bias and Methodological Quality Assessment

The methodological quality and risk of bias of the included studies were independently assessed by two reviewers (G.N. and G.M.) using the Joanna Briggs Institute critical appraisal tools appropriate to each study design [46]. Disagreements were resolved by discussion and, when necessary, by consultation with a third reviewer (E.G.). Cohort studies, comparative observational studies, registry-based studies, and case series were appraised using the corresponding JBI checklists.

Studies were classified according to their methodological design before appraisal. Descriptive procedural series without a formal comparator group were assessed using the JBI Critical Appraisal Checklist for Case Series, whereas comparative observational studies, registry-based analyses, and studies evaluating associations between procedural exposures and clinical outcomes were assessed using the JBI Critical Appraisal Checklist for Cohort Studies. Methodological appraisal was not used as an exclusion criterion; rather, it was applied to contextualize the reliability, applicability, and interpretability of the available evidence. Detailed study-level assessments are reported in Supplementary Table S2.

The assessment considered the clarity of inclusion criteria, representativeness of the study population, definition of the index procedure, completeness of follow-up, reliability of outcome measurement, reporting of procedure-related safety outcomes, and adequacy of statistical analysis when applicable.

For studies including mixed populations or mixed procedural indications, particular attention was paid to whether ECMO-specific and procedure-specific outcomes were separately extractable. Studies were not excluded solely on the basis of methodological quality; instead, quality assessment was used to contextualize the certainty and applicability of the findings.

Given the predominance of retrospective and heterogeneous observational evidence, risk-of-bias results were summarized descriptively and incorporated into the interpretation of the qualitative synthesis.

### 2.10. Data Synthesis

A qualitative synthesis was performed. Study findings were summarized narratively and organized by procedural domain. Quantitative pooling was not planned because of heterogeneity in study design, ECMO indication, ECMO configuration, procedural type, anticoagulation management, outcome definitions, and reporting of bleeding and transfusion outcomes.

Where available, numerical rates of bleeding, major bleeding, transfusion, thrombotic complications, circuit complications, oxygenator exchange, reintervention, reoperation, and mortality were extracted and reported descriptively. The synthesis was designed to emphasize clinically relevant procedural risk profiles rather than to generate a single pooled estimate across heterogeneous procedures.

Detailed study-level extraction of bleeding, transfusion, thrombotic, circuit-related, reintervention, and mortality outcomes, including comparative statistics when available, is provided in Supplementary Table S1.

## 3. Results

### 3.1. Study Selection

The PubMed/MEDLINE search identified 234 records. After title screening, 185 records were excluded because they were not procedure-specific, focused on general ECMO outcomes or anticoagulation management, involved ECMO cannulation or decannulation as the index procedure, referred to pediatric or neonatal populations, represented reviews, guidelines, consensus documents, editorials, or clearly non-eligible case reports. The remaining 49 records underwent abstract screening. Of these, 19 were excluded because they did not report procedure-specific safety outcomes, included non-separable ECMO data, described ECMO as rescue after a procedure rather than procedural support, or were case reports below the predefined eligibility threshold. Thirty PubMed/MEDLINE full texts were assessed for eligibility. Four records were excluded at full-text assessment, and 26 PubMed/MEDLINE studies were included in the qualitative synthesis.

The Scopus search identified 344 records. Twenty records were excluded before screening because they were clearly outside the scope of the review, including book chapters, conference-related records, reviews, case reports, or records not corresponding to original full-text clinical studies eligible for extraction. Ten duplicates of PubMed/MEDLINE studies were removed before screening. The remaining 314 records underwent title and abstract screening. After this phase, 51 records were considered eligible and 56 were considered potentially eligible or uncertain, resulting in 107 reports requiring full-text evaluation. After full-text assessment, 20 additional unique Scopus studies were included.

Overall, the final qualitative synthesis included 46 studies, comprising 26 studies from PubMed/MEDLINE and 20 additional unique studies from Scopus.

### 3.2. General Characteristics of Included Studies

The 46 included studies covered a broad spectrum of invasive procedures performed during adult ECMO or ECLS support. Most studies were retrospective observational cohorts, single-center or multicenter case series, registry analyses, or comparative observational studies. No randomized controlled trial specifically evaluating the safety of invasive procedures during ECMO was identified.

The included studies were organized into six procedural domains: airway, bronchoscopic, and tracheobronchial procedures; thoracic surgery and lung resections; abdominal surgery, gastrointestinal endoscopy, and decompressive laparotomy; lung transplantation and perioperative ECLS; cardiovascular, vascular, pulmonary embolism-related, and mechanical circulatory support-related procedures; and mixed non-cardiac surgical procedures.

The most frequently reported safety outcomes were bleeding, transfusion requirement, hemothorax, surgical-site bleeding, reoperation or reintervention, thrombotic complications, circuit complications, oxygenator exchange, ECMO duration, ICU or hospital length of stay, and mortality. Outcome definitions were heterogeneous across studies, and bleeding was variably reported as major bleeding, relevant hemorrhage, hemothorax, postoperative bleeding, transfusion burden, re-exploration for bleeding, or clinically relevant bleeding requiring intervention.

Detailed characteristics of the included studies are summarized in Table 1. A domain-level summary of the main procedure-specific safety outcomes is provided in Table 2. Full study-level safety outcomes, including *p*-values, odds ratios, hazard ratios, confidence intervals, and survival analyses when available, are reported in Supplementary Table S1.

Full study-level safety outcomes, including bleeding, transfusion, thrombotic and circuit-related complications, reintervention, mortality, and comparative statistics when available, are reported in Supplementary Table S1.

### 3.3. Methodological Quality and Risk-of-Bias Findings

Methodological quality assessment was performed for all 46 included studies using the Joanna Briggs Institute critical appraisal tools appropriate to study design. Twenty studies were classified as non-comparative case series, whereas 26 were classified as cohort, comparative observational, or registry-based studies.

Overall, the evidence base was dominated by retrospective observational studies and small procedural series. Case-series studies generally reported procedural indications, ECMO configuration, immediate complications, and short-term outcomes with adequate clarity; however, their interpretation was limited by small sample sizes, single-center experience, absence of comparator groups, and highly selected procedural populations. These limitations were particularly relevant in complex airway surgery, emergency thoracic surgery, decompressive laparotomy, advanced oncovascular surgery, and ECLS-to-LVAD bridging.

Among cohort and registry-based studies, the most frequent methodological concerns were confounding by indication, heterogeneous procedural indications, clinically driven selection of ECMO configuration or anticoagulation strategy, and incomplete adjustment for differences in baseline severity. Comparative studies using propensity-score methods or multivariable adjustment provided more structured evidence, particularly in non-elective thoracic surgery, emergency abdominal surgery, lung transplantation, and LVAD implantation. Nevertheless, residual confounding remained substantial because invasive procedures were generally performed in patients with severe complications or in highly selected candidates managed at experienced referral centers.

Accordingly, the available evidence primarily supports the technical achievability of selected invasive procedures during adult ECMO support and identifies clinically relevant safety signals, particularly bleeding, transfusion requirements, reintervention, hemothorax, thrombotic complications, and circuit-related events. It does not provide definitive comparative evidence that individual procedures are uniformly safe across the broader ECMO population. Detailed study-level JBI assessments are provided in Supplementary Table S2.

### 3.4. Airway, Bronchoscopic, and Tracheobronchial Procedures

Airway, bronchoscopic, and tracheobronchial procedures were evaluated in studies including high-risk bronchoscopy, whole-lung lavage, rigid bronchoscopy, bronchotracheal stenting, flexible bronchoscopy, transbronchial lung cryobiopsy, surgical and bronchoscopic tracheal procedures, complex airway surgery, and bronchoscopic cryoextraction of airway blood clots [3,4,14,15,16,24,25].

Overall, single-center airway and bronchoscopic series reported high procedural success when ECMO was used as planned support or rescue support. Stokes et al. reported eight adult patients undergoing nine airway interventions under VV-ECMO. All procedures were completed successfully, with one cannula-associated deep-vein thrombosis and no other ECMO-related complications. Survival to hospital discharge was 87.5%, and 1-year survival was 50% [3]. Meyer et al. described 14 rigid bronchoscopies in 11 patients undergoing bronchotracheal stenting under ECMO support. ECMO weaning was successful in all cases, median ECMO time was 267 min, and two local cannulation-related complications were reported [4].

Diagnostic and therapeutic bronchoscopy appeared feasible in selected patients. Redivo et al. reported 16 bronchoscopies in eight ECMO-supported patients, with no radiological worsening or hemodynamic complications. Transient desaturation occurred in one procedure, 6.25%, and moderate bleeding after transbronchial biopsy occurred in one procedure, 6.25%, controlled endoscopically [24]. Wang et al. evaluated transbronchial lung cryobiopsy in 13 VV-ECMO patients. No procedure-related death, pneumothorax, or severe bleeding occurred; however, moderate bleeding was observed in 5/13 patients, 38.5%, and was controlled with prophylactic balloon blockers. A pathological diagnosis was obtained in all patients [16].

Bronchoscopic cryoextraction for airway blood clot removal was reported by Schmidt et al. in 16 critically ill patients with pulmonary hemorrhage, 11 of whom were supported with ECMO. A total of 27 procedures were performed. Tracheobronchial obstruction was successfully relieved in all cases, no severe procedure-related complications were reported, and repeat cryoextraction was required in seven patients [25].

More complex airway surgery and registry-level data showed a higher complication burden. Onorati et al. reported 24 patients undergoing 28 procedures under planned VV-ECMO, including 11 rigid bronchoscopies and 17 airway surgeries. Major 30-day morbidity occurred in 10/24 patients, minor morbidity in 13/24, and ECMO-specific complications in four cases, including deep-vein thrombosis in three and vasoplegic syndrome in one. Thirty-day mortality was 5/24, 21% [15]. Suzuki et al., using ELSO Registry data, analyzed 269 adult ECMO patients undergoing tracheal procedures. Overall survival to discharge was 64.3%. Hemorrhagic complications occurred in 26.0%, and surgical-site bleeding occurred in 13.0%; both were associated with worse survival [14].

Taken together, airway and bronchoscopic procedures were generally feasible during ECMO in experienced centers. The lowest complication rates were reported in selected bronchoscopic and diagnostic procedures, whereas complex airway surgery and registry-level tracheal procedure data showed clinically relevant bleeding, thrombotic events, and mortality.

### 3.5. Thoracic Surgery, Chest Drainage, and Lung Resections

Thoracic surgery and thoracic interventions represented the largest procedural domain. Included procedures were non-elective thoracic surgery, lung abscess resection, emergency lobectomy or pneumonectomy, VATS, lung volume reduction surgery, tracheal sleeve pneumonectomy, chest drainage, thoracic interventions for hemothorax, and major cardiopulmonary resections [5,6,10,11,17,21,22,26,27,28,29,30,31,47,48,49].

Bleeding and reintervention were frequent in thoracic bleeding cohorts. Ried et al. analyzed 418 VV-ECMO patients and reported relevant hemorrhage in 23.2%. Thoracic bleeding occurred in 9.6% of the total cohort and accounted for 41.2% of all relevant hemorrhagic events. A thoracic operation was required in 60% of patients with thoracic bleeding, and repeated operation due to bleeding was necessary in 45.8%. ECMO duration was longer in patients with thoracic bleeding, 18.6 ± 16.8 days, *p* = 0.035, and hospital length of stay was also longer, 58 ± 50 days, *p* = 0.002. Mortality was higher in patients with thoracic bleeding than in those without bleeding complications, 52.5% versus 32.7%, *p* = 0.013 [10].

Non-elective thoracic surgery during VV-ECMO showed high but not uniformly excess bleeding risk when compared with medical VV-ECMO populations. Beyls et al. compared 44 patients receiving perioperative VV-ECMO for non-elective thoracic surgery with medically indicated VV-ECMO patients in a bicenter cohort. Hemothorax was more frequent in the perioperative group, 36% versus 2%, *p* < 0.001. Major bleeding was similar between groups, 48% versus 47%, *p* = 1.00, and thrombotic events occurred in 41% versus 32%, *p* = 0.31. Ninety-day mortality was 54% versus 51%, *p* = 0.71 before matching, with no significant difference after propensity matching, log-rank *p* = 0.95. Multivariable Cox analysis showed no association between surgical ECMO and mortality, HR 1.003, 95% CI 0.64–1.57 [11].

Emergency thoracic surgery in COVID-19 patients on VV-ECMO was associated with high mortality and bleeding burden. Almeida et al. reported nine COVID-19 patients undergoing emergency anatomical lung resection under VV-ECMO, including eight lobectomies and one pneumonectomy. ECMO weaning was successful in 4/9 patients, and in-hospital mortality was 5/9, 55.6% [17]. Zwaenepoel et al. reported 14 VATS procedures in seven COVID-19 patients on VV-ECMO, mostly for hemothorax, 85.7%. Four of seven patients died, 57.1%, including two immediate perioperative deaths due to uncontrollable bleeding. Ten circuit changes were required in six patients, 85.7%, and all three survivors required additional transarterial embolization [21].

Chest drainage during VV-ECMO was specifically evaluated by Laverty et al. in COVID-19 and non-COVID ARDS patients. Pneumothorax occurred in 44% of COVID-19 patients versus 22% of controls, OR 2.8, 95% CI 0.95–7.9, *p* = 0.09. Tube thoracostomy was performed in 36% versus 24%, OR 1.8, 95% CI 0.55–5.7. Complications after initial tube thoracostomy were significantly more frequent in COVID-19 patients, 89% versus 33%, OR 16, 95% CI 1.6–201, *p* = 0.0498 [29].

Selected planned or semi-planned thoracic procedures had more favorable procedural safety profiles. Koryllos et al. reported 24 patients undergoing major cardiopulmonary resections with intraoperative ECMO, including carinal, descending aortic, and left atrial resections. No intraoperative complications occurred, complete resection was achieved in 18/24 patients, 30-day mortality was 25%, and median survival was 12 months [5]. Schweigert et al. analyzed 127 patients undergoing non-elective major lung surgery for infectious lung abscess, including 10 supported with ECMO. No intraoperative ECMO-associated complications were reported, and mortality was 1/10 in the ECMO group versus 16/117 in the non-ECMO group. ECMO use was not associated with mortality, OR 0.70, 95% CI 0.08–5.91, *p* = 0.74 [6].

Other thoracic series confirmed feasibility but showed variable complication rates. Huang et al. reported 22 patients receiving perioperative ECMO during thoracic surgery; severe hemorrhage occurred in 13.6%, successful decannulation in 90.9%, and survival to discharge in 77.2%. Survival did not differ significantly between preoperative and postoperative ECMO, *p* = 0.135, or between VV-ECMO and VA-ECMO, *p* = 0.550 [27]. Zhang et al. reported 15 ECMO-assisted complex thoracic surgery patients, with hemorrhage in 26.7%, infection in 33.3%, acute hepatic dysfunction in 33.3%, venous thrombosis in 26.7%, hospital mortality of 6.7%, and 1-year survival of 86% [30]. Kim et al. reported 63 high-risk thoracic surgery patients supported with ECMO. Intraoperative arrest occurred in 17.5% and was independently associated with mortality, OR 24.44, 95% CI 1.82–327.60, *p* = 0.016; age was also independently associated with mortality, OR 7.47, 95% CI 1.17–47.85, *p* = 0.034 [28].

Akil et al. compared non-intubated versus intubated VATS lung volume reduction surgery in 92 patients, all supported with low-flow VV-ECLS. Chest tubes were removed earlier in the non-intubated group, 5 ± 1 versus 8 ± 1 days, *p* < 0.02. ICU stay was shorter, 4 ± 1 versus 8 ± 2 days, *p* = 0.04, and hospital stay was shorter, 6 ± 2 versus 10 ± 4 days, *p* = 0.01. Ninety-day mortality was 3% versus 7%, and conversion to general anesthesia was required in one patient [22].

Overall, thoracic procedures during ECMO were technically achievable, but their safety profile was highly procedure-dependent. Emergency thoracic bleeding, hemothorax, COVID-19-related thoracic complications, and non-elective surgery were associated with substantial bleeding, reintervention, and mortality burdens; therefore, technical completion in these settings should not be interpreted as evidence of favorable procedural safety. In contrast, selected planned thoracic procedures showed more acceptable safety profiles in specialized centers.

### 3.6. Abdominal Surgery, Gastrointestinal Endoscopy, and Decompressive Laparotomy

Abdominal procedures included emergency abdominal surgery, emergency laparotomy, abdominal exploration, decompressive laparotomy for abdominal compartment syndrome, open abdomen therapy, and endoscopic management of gastrointestinal bleeding during ECMO support [7,13,18,32,33,34,35,48].

Emergency abdominal surgery was associated with a high bleeding and transfusion burden. Taieb et al. compared 35 ECMO patients undergoing emergency abdominal surgery with 42 non-ECMO ICU controls. Postoperative transfusion was more frequent in ECMO patients, 77% versus 40%. Median packed red blood cell transfusion was 13 units, IQR 6–22, versus 3 units, IQR 0–5; fresh frozen plasma was 9 units, IQR 3–17, versus 0 units, IQR 0–4; and platelet transfusion was 12 units, IQR 3–22, versus 0 units, IQR 0–8; all *p* < 0.001. Reintervention for hemorrhage was required in 20% versus 2%, *p* = 0.02. In multivariable analysis, ECMO was independently associated with bleeding, OR 5.6, 95% CI 2.0–15.4, *p* = 0.001. ICU mortality was higher in ECMO patients, 69% versus 33%, *p* = 0.003, whereas perioperative mortality was similar, 11% versus 12% [7].

Abdominal exploration during ECMO was also associated with high mortality, largely reflecting underlying disease severity. Jena et al. analyzed 56 abdominal explorations among 1386 ECMO-supported patients, corresponding to 4% of the ECMO cohort. Indications included mesenteric ischemia, intra-abdominal hemorrhage, abdominal compartment syndrome, and bowel perforation. In-hospital mortality was 57%. Non-survivors had higher APACHE II scores, 17 versus 9, *p* < 0.001, higher SOFA scores, 8 versus 4, *p* < 0.01, and higher pre-ECMO lactate levels, 11 versus 3 mmol/L, *p* < 0.001. Operative findings suggested that most ischemic injuries represented watershed hypoperfusion related to cardiogenic shock and ECMO physiology, whereas true thromboembolic occlusion was uncommon [13].

Decompressive laparotomy for abdominal compartment syndrome was evaluated in several studies. Glowka et al. reported 11 patients who developed abdominal compartment syndrome and underwent decompressive laparotomy among 175 ECMO patients. Survival to hospital discharge was 27.3%, with mortality of 72.7%. Mortality did not significantly differ between patients with decompressive laparotomy and those without it, 73% versus 65%, *p* = 0.749. Risk factors associated with mortality included age, *p* = 0.032, Charlson comorbidity index >1, *p* = 0.004, SAPS II ≥42 at ICU admission, *p* = 0.013, and SAPS II ≥44 at ECMO initiation, *p* = 0.004 [33].

Lubnow et al. identified abdominal compartment syndrome in 47 of 1643 ECMO patients, 2.9%, with a higher prevalence among resuscitated ECMO patients, 4.2%. Decompressive laparotomy decreased intra-abdominal pressure and improved ventilation, while vasopressor requirements and lactate stabilized within 24 h. Patients undergoing decompressive laparotomy were more severely ill, with SOFA score at ICU admission 18, IQR 15–20, versus 16, IQR 13–17, *p* = 0.048. Survival was 11% in the decompressive laparotomy group versus 14% in the non-laparotomy group, *p* = 1.000 [18].

Open abdomen therapy was described by Schulz et al. in eight patients among 421 ECMO cases, 1.9%. Median duration of open abdomen therapy was 17 days, and median ICU length of stay was 42 days. The median number of surgical procedures and negative-pressure wound therapy dressing changes was seven. Surgical revision was required in 3/8 patients. Overall mortality was 50%, and fascial closure was achieved in 75%. Abdominal packing due to severe bleeding was reported in all deceased patients and only once among survivors, suggesting a potential association with poor outcome [34].

McCann et al. reported emergency laparotomy in 3.7% of ECMO patients with severe respiratory failure. Survival to hospital discharge was 31%. Major hemorrhage was uncommon, but emergency oxygenator change was commonly required. Duration of ECMO support and ICU stay after decannulation did not differ between patients requiring laparotomy and those not requiring laparotomy [32]. Boulos et al. described nine VV-ECMO patients undergoing early decompressive laparotomy for intra-abdominal hypertension or abdominal compartment syndrome. Decompression improved ECMO flow, oxygenation, and pulmonary compliance; no major surgical or bleeding complications were reported, and survival to discharge was 56% [48].

Endoscopic management of gastrointestinal bleeding appeared feasible and effective in selected patients. Amata et al. analyzed 134 VV-ECMO patients and identified gastrointestinal bleeding in 14 patients, 10.4%. Active bleeding was found in 11/14 patients, 79%. Endoscopic therapy achieved complete bleeding control in all active cases. ECMO duration was longer in patients with gastrointestinal bleeding, 19.5 days, IQR 15–36, versus 13.5 days, IQR 8–25, *p* = 0.01. No patient died from fatal gastrointestinal bleeding, and mortality did not significantly differ between patients with and without gastrointestinal bleeding [35].

Overall, abdominal surgery during ECMO was associated with high mortality and substantial bleeding risk, particularly in emergency abdominal surgery and abdominal exploration. Decompressive laparotomy and open abdomen therapy were technically performable in selected patients but were undertaken in the setting of severe multiorgan failure and remained associated with poor outcomes. In contrast, endoscopic management of gastrointestinal bleeding achieved complete hemostasis in selected VV-ECMO patients.

### 3.7. Lung Transplantation and Perioperative ECLS

Lung transplantation represented a distinct procedural domain because ECMO or ECLS was frequently used intraoperatively or perioperatively as planned support rather than only as rescue therapy. Included studies evaluated intraoperative ECMO, perioperative ECLS-related complications, central versus peripheral ECMO, heparin-free ECMO strategies, intraoperative anticoagulation, extended central VA-ECMO, delayed chest closure, and surgical approach during bilateral lung transplantation [8,9,12,19,20,23,36].

Ius et al. analyzed 595 lung transplant recipients, of whom 170, 29%, required intraoperative ECMO. Ninety-five patients received ECMO based on an a priori indication, whereas 75 required ECMO intraoperatively. Patients requiring ECMO had a higher preoperative risk profile and more postoperative complications than patients transplanted without ECMO. Overall survival at 1 year was 93% in patients without ECMO, 83% in patients with a priori ECMO, and 82% in patients requiring intraoperative ECMO; 4-year survival was 73%, 68%, and 69%, respectively, with no statistically significant difference among groups, *p* = 0.11. Intraoperative ECMO did not emerge as a risk factor for in-hospital mortality or mortality after hospital discharge [8].

Orlitová et al. reviewed 703 lung transplantations, with perioperative ECLS used in 156 patients, 22%. Among supported patients, VA support was used in 120/156, 77%, VV-ECMO in 25/156, 16%, and cardiopulmonary bypass in 11/156, 7%. At least one ECLS-related complication occurred in 104/156 patients, 67%. The most common complications were hemothorax in 39/156, 25%, continuous renal replacement therapy in 30/156, 19%, and thromboembolism in 22/156, 14%. Thirty-day mortality was 6% [9].

Qi et al. evaluated 324 lung transplantation patients managed with early perioperative heparin-free ECMO, including 251 VV-ECMO, 40 VA-ECMO, and 33 VV-A ECMO patients. The VV-ECMO group had the lowest intraoperative bleeding, 24 h thoracic drainage, and transfusion demand. Vein thrombosis occurred in 30.2% of patients within 10 days postoperatively or within one week after ECMO withdrawal, with no significant difference among ECMO configurations. Except for one acute myocardial infarction, no other serious thrombotic events occurred, and no thrombosis-related deaths were reported. Intraoperative bleeding and 24 h thoracic drainage were independent predictors of 1-year survival [12].

Vajter et al. analyzed 109 lung transplantation patients who underwent central VA-ECMO with successful intraoperative ECMO weaning. Lower unfractionated heparin doses during intraoperative ECMO anticoagulation were associated with reduced blood loss and blood product consumption. Lower heparin dosing was also associated with fewer surgical revisions for hemothorax, without patient- or circuit-related thrombotic complications [19].

Ruszel et al. reviewed 77 lung transplantations, of which 40, 51%, required intraoperative extracorporeal assistance. Thirty-two patients received ECMO and eight received cardiopulmonary bypass. In the ECMO group, 14/32 patients, 44%, underwent peripheral cannulation and 18/32, 56%, central cannulation. Acute kidney injury and thromboembolic complications occurred more frequently among patients requiring support, with *p* = 0.005 and *p* = 0.02, respectively. Central ECMO was associated with 30-day, 1-year, 3-year, and 5-year survival rates of 78%, 66%, 66%, and 66%, respectively; corresponding rates were 50%, 41%, 41%, and 33% for peripheral ECMO, and 75%, 50%, 50%, and 38% for cardiopulmonary bypass [20].

Kawashima et al. evaluated 20 adult patients with pulmonary arterial hypertension undergoing lung transplantation. Seventeen patients were managed with extended postoperative central VA-ECMO and delayed chest closure. Median ECMO duration was 4 days. Hemothorax evacuation was required in 9/20 patients, 45.0%. No thrombotic complications were reported. Median ICU length of stay was 16 days, and median hospital length of stay was 52.5 days. Ninety-day and 1-year survival were both 100% [23].

Park et al. compared video-assisted thoracic surgery with clamshell incision in 136 bilateral lung transplantations performed with ECMO support. The VATS group had shorter operative time, 319 versus 417 min, *p* < 0.001, and lower blood loss, 832 versus 2789 mL, *p* < 0.001. The VATS group also required fewer transfusions. Sternal wound complications and airway interventions occurred exclusively in the clamshell group. Pulmonary function was significantly higher in the VATS group at 1 month postoperatively, with FEV1 85.79 ± 18.00 versus 67.25 ± 22.55, *p* < 0.001, and FVC 73.42 ± 14.14 versus 60.00 ± 17.81, *p* < 0.001. Differences persisted at 12 months, with FEV1 *p* = 0.05 and FVC *p* = 0.04 [36].

Overall, lung transplantation studies showed that ECMO and ECLS can be integrated into intraoperative and perioperative transplant pathways, but bleeding, hemothorax, transfusion burden, thromboembolism, and renal replacement therapy remained frequent safety outcomes. Surgical approach, ECMO configuration, and anticoagulation strategy appeared to influence bleeding and transfusion requirements.

### 3.8. Cardiovascular, Vascular, Pulmonary Embolism, and Mechanical Circulatory Support-Related Procedures

Cardiovascular, vascular, pulmonary embolism-related, and mechanical circulatory support-related procedures included durable LVAD implantation after ECLS bridge, LVAD implantation on ECLS in patients with heparin-induced thrombocytopenia antibodies, robot-assisted coronary artery bypass with peripheral ECMO, thoracoabdominal aortic replacement combined with oncologic surgery, and invasive treatment strategies for critical pulmonary embolism requiring ECMO [37,38,39,40,41,42,43].

Zubarevich et al. analyzed 35 patients in acute cardiogenic shock bridged with ECLS to durable LVAD implantation. Median ECLS duration was 7 days, IQR 5.0–13.0. Re-sternotomy for bleeding occurred in 42.9% and early LVAD thrombosis in 2.9%. In-hospital, 30-day, 6-month, and 1-year survival were 65.6%, 75.9%, 69.2%, and 62.7%, respectively. Severe right heart failure occurred in 22.9%, acute kidney injury requiring dialysis in 68.6%, and respiratory failure in 77.1% [37].

Potapov et al. evaluated 531 patients undergoing durable LVAD implantation after VA-ECLS using data from the ECLS-VAD registry. In the propensity score-adjusted cohort, 324 patients underwent full sternotomy and 39 underwent less invasive surgery. Median surgical duration was 236 min in the full sternotomy group versus 263 min in the less invasive surgery group, *p* = 0.289. Median chest tube output during the first 24 h was similar between groups. Patients undergoing full sternotomy required more blood products during the first 24 postoperative hours, median 16 versus 12 units, *p* = 0.033. Revision for bleeding was more frequent after full sternotomy, 35.5% versus 15.4%, *p* = 0.016. No stroke occurred in the less invasive surgery group during the first 30 days, compared with 7.4% in the full sternotomy group. Thirty-day and 1-year survival were similar between groups [38].

Sorensen et al. compared patients undergoing durable LVAD implantation via left thoracotomy/hemisternotomy according to temporary mechanical circulatory support. Among 83 patients, 50 had no temporary support, 22 received intra-aortic balloon pump, and 11 were bridged with ECMO. All ECMO patients were INTERMACS profile 1. Operative outcomes were similar across groups, although patients bridged with ECMO required more concomitant procedures and more red-cell transfusions. ICU and hospital length of stay, inotrope duration, bleeding, stroke, and infection rates did not differ significantly among groups. Three- and 12-month survival were 81% and 81% in the ECMO group, compared with 94% and 86% in patients without temporary support and 100% and 88% in IABP patients, *p* = 0.45 [39].

Ljajikj et al. compared low-dose bivalirudin anticoagulation with heparin during LVAD implantation on ECLS in patients with heparin-induced thrombocytopenia antibodies. Twenty-one patients received bivalirudin and 36 received heparin. Early surgical re-exploration within seven days for persistent hemorrhage or cardiac tamponade occurred in 19% of the bivalirudin group and 16.7% of the heparin group, OR 1.18, 95% CI 0.29–4.76, *p* = 0.820. Propensity score matching also showed no significant difference, *p* = 0.455. Secondary outcomes, including delayed chest closure, stroke, intracranial bleeding, delayed re-thoracotomy, and mortality up to one year, were comparable between groups [40].

Patel et al. reported 45 high-risk patients undergoing robot-assisted coronary artery bypass with non-emergency intraoperative peripheral ECMO support. Indications included inability to tolerate single-lung ventilation in 38%, ejection fraction below 35% in 38%, inadequate internal thoracic artery exposure in 53%, critical coronary artery disease in 36%, and hemodynamic instability after anesthesia induction in 7%. Up to 30 days, there were no strokes, myocardial infarctions, or access-vessel complications. One patient, 2.2%, died from a non-cardiac cause. One redo-CAB patient, 2.2%, required sternotomy to locate the target vessel. Thirty-four patients, 75.6%, were extubated within six hours after surgery [41].

Lutz et al. described five patients undergoing thoracoabdominal aortic replacement with rerouting of visceral and renal vessels combined with curative oncologic surgery, using ECMO as partial left-heart bypass. Early technical success was 100%, and all patients underwent complete tumor resection with negative margins. However, all five patients required surgical revision. Revision indications included liquor leak in two patients, hematoma in three, bypass revision in one, bleeding in one, and biliary leak in one. During a mean follow-up of 47 months, primary patency of aortic reconstructions and arterial bypasses was 100%, and no patient developed recurrent malignant disease [42].

Takabayashi et al. evaluated 76 patients with critical acute pulmonary embolism requiring ECMO in the COMMAND VTE Registry-2. Thirty-day mortality was 30.3%, and major bleeding at 30 days occurred in 54.0%. Procedure-site or surgery-related bleeding accounted for 22.4% of bleeding events. Mortality differed by treatment strategy: 6.3% in the surgical intervention group, 43.8% in the catheter intervention group, 25.0% in the systemic thrombolysis group, and 39.3% in the anticoagulation-only group [43].

Overall, cardiovascular, vascular, pulmonary embolism-related, and mechanical circulatory support-related procedures were technically achievable in selected ECMO-supported patients, but bleeding, transfusion requirement, surgical revision, major bleeding, and organ dysfunction were frequent. In rescue settings such as critical pulmonary embolism, technical intervention should not be equated with low procedural risk or improved comparative outcome. Less invasive surgical approaches and individualized anticoagulation strategies were associated with lower bleeding-related morbidity in some cohorts.

### 3.9. Mixed Non-Cardiac Surgical Procedures

Two studies evaluated heterogeneous non-cardiac procedures performed during extracorporeal support [1,2].

Fierro et al. described 14 VV-ECMO patients undergoing 21 major non-cardiac surgical procedures. Intraoperative blood product use was frequent: red blood cells were administered in 52.4% of procedures, fresh frozen plasma in 23.8%, and platelets in 28.6%. Hemoglobin remained stable across the perioperative period, with mean values of 9.5 g/dL preoperatively, 9.7 g/dL immediately postoperatively, and 9.5 g/dL 24 h after surgery. Intraoperative oxygenation and hemodynamic instability were common: SpO_2_ below 90% occurred in 50% of procedures, SpO_2_ below 80% in 15%, and vasopressors were used in 66.7%. One-year survival after ECMO cannulation was 50% [1].

Surman et al. reviewed 12 non-cardiac surgical cases performed with CPB or ECMO support, including three ECMO cases and nine CPB/standby or support cases. Procedures included thoracic, renal, and tracheal surgery. Severe hemorrhage occurred in 3/12 cases. No direct CPB- or ECMO-related complications were reported. Prolonged ECMO support and ICU stay occurred in seven cases. Mortality and complication data were not separately reported for ECMO-only cases [2].

These mixed cohorts suggest that non-cardiac surgery during ECMO is possible but often requires frequent intraoperative respiratory, hemodynamic, and transfusion support. The limited sample sizes and mixed ECMO/CPB populations restrict procedure-specific conclusions.

For rapid clinical reference, Table 3 summarizes the procedural settings associated with the greatest reported safety concerns across the included literature and classifies each setting according to its predominant procedural phenotype: planned procedural ECMO, reactive intervention during ECMO-associated critical illness, or mixed/context-dependent use. Because of substantial heterogeneity in study design, patient selection, ECMO configuration, procedural urgency, anticoagulation management, and outcome definitions, this summary should not be interpreted as a formal comparative ranking of procedural risk. Rather, it identifies the scenarios in which bleeding, transfusion requirements, reintervention, thrombotic events, circuit complications, and mortality were most consistently reported.

### 3.10. Overall Synthesis of Safety Outcomes

Across the 46 included studies, the safety profile of invasive procedures during adult ECMO varied markedly by procedural domain, urgency, baseline disease severity, anticoagulation strategy, and institutional expertise. Bleeding was the most frequently reported safety outcome, but definitions were heterogeneous. Events were reported as intraoperative blood loss, postoperative hemorrhage, hemothorax, surgical-site bleeding, major bleeding, transfusion burden, re-exploration for bleeding, or clinically relevant bleeding requiring endoscopic, radiologic, or surgical intervention.

The highest bleeding and transfusion burden was observed in emergency abdominal surgery, thoracic bleeding, non-elective thoracic surgery, COVID-19-related thoracic interventions, pulmonary embolism interventions, and selected lung transplantation cohorts. In emergency abdominal surgery, ECMO was independently associated with bleeding, OR 5.6, 95% CI 2.0–15.4, *p* = 0.001 [7]. In thoracic bleeding during VV-ECMO, mortality was significantly higher in patients with thoracic bleeding than in those without bleeding complications, 52.5% versus 32.7%, *p* = 0.013 [10]. In non-elective thoracic surgery, major bleeding was common but not significantly different between perioperative and medical VV-ECMO groups, 48% versus 47%, *p* = 1.00, whereas hemothorax was more frequent in surgical ECMO patients, 36% versus 2%, *p* < 0.001 [11].

Airway and bronchoscopic procedures generally showed high procedural success and acceptable safety in selected cohorts. However, registry-level tracheal procedure data demonstrated hemorrhagic complications in 26.0% and surgical-site bleeding in 13.0%, both associated with worse survival [14]. Transbronchial lung cryobiopsy during VV-ECMO was not associated with procedure-related death, pneumothorax, or severe bleeding, but moderate bleeding occurred in 38.5% and required prophylactic balloon control [16].

Abdominal compartment syndrome and abdominal exploration were associated with high mortality, reflecting severe multiorgan failure. Mortality after decompressive laparotomy for abdominal compartment syndrome ranged from 72.7% in one cohort to similarly poor survival in smaller ACS subgroups [18,33]. Conversely, endoscopic treatment of gastrointestinal bleeding during VV-ECMO achieved complete control of active bleeding in selected patients and was not associated with fatal gastrointestinal bleeding [35].

In lung transplantation, bleeding, hemothorax, thromboembolism, and transfusion requirement remained central safety outcomes. ECLS-related complications occurred in 67% of supported lung transplant patients in one cohort, including hemothorax in 25% and thromboembolism in 14% [9]. Heparin-free or lower-heparin anticoagulation strategies appeared feasible in selected transplant cohorts, with no thrombosis-related deaths in one heparin-free series and no patient- or circuit-related thrombotic complications in a lower-heparin intraoperative VA-ECMO cohort [12,19].

Thrombotic and circuit-related complications were less consistently reported than bleeding outcomes. Reported events included cannula-associated DVT, DVT during planned VV-ECMO for airway surgery, venous thrombosis during thoracic surgery, thromboembolism after lung transplantation with ECLS, vein thrombosis after heparin-free ECMO in lung transplantation, and circuit changes during VATS in COVID-19 VV-ECMO patients [3,9,12,15,21,30].

Overall, selected invasive procedures during adult ECMO were technically achievable in experienced centers. However, procedure-specific risk varied substantially. Emergency abdominal surgery, decompressive laparotomy for abdominal compartment syndrome, thoracic bleeding, COVID-19-related thoracic interventions, and critical pulmonary embolism procedures were technically performable but showed the highest bleeding, transfusion, reintervention, and mortality burdens. Selected airway, bronchoscopic, transplantation, planned thoracic, cardiovascular, and mechanical circulatory support-related procedures showed more acceptable procedural safety when performed with multidisciplinary planning and tailored anticoagulation management.

## 4. Discussion

### 4.1. Main Findings

Detailed study-level outcome data are provided in Supplementary Table S1; therefore, this discussion focuses on the interpretation of the main procedure-specific safety patterns.

This systematic review shows that invasive procedures during adult ECMO may be technically achievable in selected patients, particularly when performed in experienced centers with multidisciplinary expertise. However, technical achievability should not be interpreted as uniform procedural safety or improved clinical benefit. The risk profile varies substantially according to procedural domain, urgency, baseline disease severity, anticoagulation strategy, and the underlying indication for ECMO.

Airway and bronchoscopic procedures generally showed high procedural success in selected cohorts, especially when ECMO was used as planned respiratory support. In contrast, thoracic bleeding, emergency abdominal surgery, abdominal compartment syndrome, COVID-19-related thoracic interventions, and critical pulmonary embolism requiring ECMO were associated with substantially higher bleeding, reintervention, circuit-related complication, and mortality burdens [3,7,10,11,14,16,21,43].

Bleeding was the dominant safety concern across the included studies. Nevertheless, bleeding was inconsistently defined, being reported as major bleeding, relevant hemorrhage, hemothorax, surgical-site bleeding, transfusion requirement, reoperation for bleeding, or clinically relevant bleeding requiring intervention. This heterogeneity prevented quantitative pooling but supports a procedure-centered qualitative synthesis.

### 4.2. Planned Procedural ECMO Versus Reactive Invasive Procedures During ECMO Support

A central interpretative distinction emerging from this review is that invasive procedures during ECMO do not represent a single clinical scenario. In planned procedural ECMO, extracorporeal support is deployed prospectively or perioperatively to facilitate a procedure that might otherwise be technically unsafe or physiologically intolerable, as in complex airway interventions, selected thoracic resections, lung transplantation, minimally invasive LVAD implantation, or robot-assisted coronary revascularization [3,4,5,6,8,12,15,16,19,22,23,27,30,36,38,41]. In these settings, ECMO primarily functions as a controlled physiologic platform that may maintain gas exchange or circulatory stability while the index procedure is performed.

By contrast, reactive invasive procedures during ECMO-associated critical illness are undertaken because of a complication or deterioration arising during an already severe clinical course, including thoracic bleeding or hemothorax requiring surgical intervention, emergency abdominal exploration, decompressive laparotomy for abdominal compartment syndrome, gastrointestinal bleeding requiring endoscopic treatment, or emergency thoracic procedures in prolonged COVID-19 VV-ECMO support [7,10,13,17,18,21,26,29,32,33,34,35,48]. In these situations, bleeding, transfusion burden, circuit complications, reintervention, and mortality reflect not only procedural trauma but also the severity of the precipitating complication and the underlying critical illness.

This distinction is relevant when interpreting apparent procedural success. In planned settings, ECMO may increase technical operability and allow completion of highly complex interventions; however, the current evidence does not demonstrate that expanded technical feasibility necessarily translates into improved long-term survival, oncological outcomes, or patient-centered benefit. Conversely, unfavorable outcomes after reactive procedures should not be attributed solely to the intervention itself. Planned versus reactive procedural phenotype therefore provides a clinically useful interpretative framework, but not a formal comparative risk classification.

### 4.3. Procedure-Specific Risk Patterns

The findings of this review suggest that ECMO status alone should not be considered an absolute barrier to invasive procedures. Rather, the procedural indication, urgency, expected bleeding risk, reversibility of the underlying disease, and local expertise should guide decision-making.

Airway and bronchoscopic interventions appeared among the more favorable procedural domains. High-risk bronchoscopy, bronchotracheal stenting, transbronchial lung cryobiopsy, and bronchoscopic cryoextraction were generally feasible in selected patients, with high procedural success and limited severe procedure-related bleeding in single-center series [3,4,16,24,25]. However, registry-level tracheal procedure data demonstrated that hemorrhagic complications and surgical-site bleeding may substantially affect outcomes, indicating that airway procedures should not be considered uniformly low-risk [14].

Thoracic procedures showed the broadest variability. Planned or semi-planned thoracic operations, including selected major cardiopulmonary resections, lung volume reduction surgery, and perioperative ECMO-assisted thoracic surgery, were feasible in specialized centers [5,22,27,30]. Conversely, thoracic bleeding, hemothorax, COVID-19-related VATS, and non-elective thoracic surgery were associated with frequent reintervention, circuit changes, and increased mortality [10,11,21,29]. This distinction is clinically important because the risk of a planned thoracic procedure supported by ECMO differs markedly from that of surgery performed to manage bleeding or severe infectious complications during prolonged ECMO. Importantly, the particularly high bleeding and circuit-intervention burden reported in COVID-19 VV-ECMO thoracic cohorts should be interpreted as context-specific. These findings arose in patients undergoing emergency intervention during severe COVID-19-related critical illness and prolonged extracorporeal support, a setting potentially characterized by additional inflammatory, endothelial, and thrombo-inflammatory disturbances. Therefore, these outcomes should not be directly generalized to non-COVID ECMO populations undergoing thoracic procedures [17,21,29].

Abdominal procedures represented one of the highest-risk domains. Emergency abdominal surgery and abdominal exploration were associated with high transfusion requirements and mortality, likely reflecting both surgical risk and severe underlying shock, mesenteric ischemia, abdominal compartment syndrome, and multiorgan failure [7,13,18,33]. Decompressive laparotomy may improve intra-abdominal pressure and physiological parameters in selected patients, but survival remained poor in most series [18,33]. In contrast, endoscopic management of gastrointestinal bleeding achieved complete hemostasis in selected VV-ECMO patients, suggesting that less invasive approaches may be preferable when technically feasible [35].

Lung transplantation was a distinct procedural field because ECMO or ECLS was often part of the planned intraoperative or perioperative strategy rather than a purely rescue intervention. In this domain, bleeding, hemothorax, transfusion requirement, thromboembolism, and renal replacement therapy remained relevant safety outcomes. Surgical approach and anticoagulation strategy appeared to influence bleeding and transfusion burden, particularly in studies evaluating heparin-free ECMO, lower-dose heparin, and VATS versus clamshell incision [8,9,12,19,36].

### 4.4. Bleeding and Transfusion Burden

The highest bleeding and transfusion burden was observed in emergency abdominal surgery, thoracic bleeding, non-elective thoracic surgery, pulmonary embolism interventions, and selected lung transplantation cohorts [7,10,11,23,36,43]. In emergency abdominal surgery, ECMO was independently associated with bleeding and was accompanied by markedly higher red blood cell, fresh frozen plasma, and platelet requirements [7]. This finding suggests that bleeding risk is not only a consequence of baseline critical illness but may be amplified by the combination of ECMO-associated coagulopathy, anticoagulation exposure, and surgical trauma.

Thoracic bleeding was similarly prognostically relevant. Patients with thoracic bleeding during VV-ECMO had longer ECMO duration, longer hospitalization, more frequent thoracic operations and repeat operations, and higher mortality than patients without bleeding complications [10]. In non-elective thoracic surgery, major bleeding was common, while hemothorax was substantially more frequent among surgical ECMO patients than among medical VV-ECMO patients [11].

In lung transplantation, hemothorax and transfusion burden were recurrent safety outcomes. Lower heparin exposure and less invasive surgical approaches were associated with lower blood loss, fewer transfusions, or fewer revisions for hemothorax in selected cohorts [19,36]. These findings are clinically relevant, but they should be interpreted cautiously because they derive from observational studies with potential selection bias.

### 4.5. Thrombotic and Circuit-Related Complications

Thrombotic and circuit-related complications were less consistently reported than bleeding outcomes. When available, reported events included deep-vein thrombosis, cannula-associated thrombosis, venous thrombosis, thromboembolism, stroke, circuit clotting, oxygenator exchange, and circuit changes [3,9,12,15,21,30].

The data highlight the need to balance bleeding prevention with thrombosis prevention. Temporary reduction or interruption of anticoagulation was used in several procedural settings, but thrombotic events were not absent. DVT was reported in airway intervention cohorts, venous thrombosis occurred in thoracic surgery and heparin-free lung transplantation cohorts, thromboembolism was reported after lung transplantation with ECLS, and circuit changes were frequent in COVID-19 patients undergoing VATS on VV-ECMO [3,9,12,15,21,30].

Current evidence does not support a single anticoagulation strategy for all invasive procedures during ECMO. Instead, anticoagulation should be individualized according to ECMO configuration, procedural bleeding risk, circuit performance, patient coagulation profile, thrombosis history, and expected duration of anticoagulation interruption [12,19,21,40].

### 4.6. Anticoagulation Management

Anticoagulation management was variably reported and rarely standardized. Some studies described unfractionated heparin protocols, heparin-free strategies, lower-dose heparin regimens, temporary perioperative discontinuation, or alternative anticoagulants such as bivalirudin [12,19,21,40].

The lung transplantation literature provides useful insights. Heparin-free perioperative ECMO was feasible in a large cohort and was not associated with thrombosis-related deaths, although vein thrombosis remained frequent [12]. Lower-dose unfractionated heparin during intraoperative ECMO was associated with lower blood loss, reduced blood product consumption, and fewer revisions for hemothorax, without reported patient- or circuit-related thrombotic complications [19].

In the mechanical circulatory support setting, bivalirudin was evaluated in patients with heparin-induced thrombocytopenia antibodies undergoing LVAD implantation on ECLS. Early re-exploration for bleeding or tamponade was similar between bivalirudin and heparin groups, supporting feasibility in selected patients, although superiority was not demonstrated [40]. Additional evidence on bivalirudin in critically ill COVID-19 patients provides useful contextual information on anticoagulation in prothrombotic and bleeding-prone critical illness, but it should not be interpreted as direct evidence for procedural safety during ECMO [50].

An additional limitation of the available evidence is that peri-procedural anticoagulation monitoring was not reported uniformly across studies. In clinical practice, monitoring may include activated clotting time (ACT), activated partial thromboplastin time (aPTT), anti-factor Xa activity, and/or viscoelastic testing such as thromboelastography (TEG) or rotational thromboelastometry (ROTEM). However, the included literature inconsistently reported monitoring modality, target range, timing relative to the index procedure, criteria for interruption or dose reduction, and timing of anticoagulation resumption. This variability limits comparisons among heparin-free, reduced-heparin, and bivalirudin-based strategies and supports the need for standardized reporting of anticoagulant dose, monitoring targets, circuit surveillance, and peri-procedural restart protocols in future procedural ECMO studies.

### 4.7. Clinical Implications

The decision to perform an invasive procedure during ECMO should be procedure-specific rather than based only on the presence of ECMO support. Selected bronchoscopic, airway, transplant, planned thoracic, cardiovascular, and mechanical circulatory support-related procedures may be reasonable in experienced centers. Conversely, emergency abdominal surgery, thoracic bleeding, abdominal compartment syndrome, and COVID-19-related thoracic interventions should be considered high-risk scenarios requiring careful multidisciplinary evaluation [7,10,13,18,21,29].

Procedural planning should include explicit assessment of anticoagulation strategy, transfusion thresholds, hemostatic optimization, circuit performance, oxygenator reserve, blood product availability, surgical or interventional radiology backup, and contingency planning for circuit or oxygenator exchange. For procedures with high bleeding risk, a predefined plan for anticoagulation interruption and restart is essential. For procedures with prolonged anticoagulation interruption or high thrombotic risk, intensified circuit monitoring should be considered.

Whenever clinically appropriate, less invasive approaches may reduce procedural trauma and bleeding burden. Examples include endoscopic hemostasis for gastrointestinal bleeding, bronchoscopic management of airway obstruction or clot removal, selected VATS approaches, and less invasive LVAD implantation strategies [16,25,35,36,38]. However, these approaches require careful patient selection and should not be generalized to unstable patients in whom rapid source control is required.

Implementation of procedural ECMO also depends on institutional resources and organization. Programs undertaking planned or emergency invasive procedures during ECMO should have established multidisciplinary pathways involving ECMO intensivists, anesthesiologists, surgeons or interventional specialists, perfusion personnel, transfusion support, and immediate access to imaging and rescue procedures when required. In addition to cannulation and circuit expertise, safe implementation requires protocols for hemostatic assessment, anticoagulation interruption and resumption, circuit surveillance, blood-product availability, management of massive bleeding, and escalation to surgical or interventional hemostasis. These requirements underline the resource intensity of procedural ECMO and may limit its generalizability and scalability outside experienced centers.

Patient selection should therefore consider not only whether ECMO can make an intervention technically achievable, but also whether the expected procedural benefit is proportionate to the burden of support and the patient’s underlying prognosis. Relevant considerations include reversibility of the acute disease, frailty and comorbidity burden, oncological prognosis when applicable, anticipated duration of postoperative support, and the availability of a realistic bridge-to-recovery, bridge-to-transplantation, or bridge-to-durable-support strategy. In prolonged postoperative support, advanced oncological disease, or situations in which no meaningful exit strategy remains available, repeated multidisciplinary reassessment of goals of care and proportionality is essential to avoid technically successful but clinically non-beneficial interventions.

### 4.8. Relationship with Existing Literature

Previous publications have addressed selected applications of extracorporeal support in operative settings. Suk et al. reviewed the use of ECMO in thoracic surgery excluding lung transplantation; Rocco discussed elective perioperative VV-ECMO for complex central airway surgery as a transition from rescue therapy to a proactive safety net; and Miller et al. reviewed ECMO across the perioperative phases of operative trauma care [51,52,53]. These publications establish that procedural ECMO is an evolving field, but they remain focused on individual procedural areas or specific perioperative populations.

The added value of the present systematic review lies in integrating, within a single adult procedure-centered synthesis, both planned procedural ECMO and reactive invasive interventions performed during ECMO-associated critical illness across airway and bronchoscopic procedures, thoracic surgery, abdominal surgery, lung transplantation, cardiovascular and vascular procedures, pulmonary embolism interventions, and mechanical circulatory support-related surgery. This integrated approach permits comparison of recurring safety signals, including bleeding, transfusion burden, thrombotic and circuit-related complications, and reintervention, while avoiding the assumption that improved technical operability necessarily translates into improved long-term clinical outcomes.

Tracheostomy during ECMO has been specifically addressed in a dedicated systematic review and was therefore excluded from this review to avoid duplication [44]. The current synthesis complements that evidence by showing that other airway and tracheobronchial procedures may also be technically achievable during ECMO, but can carry clinically relevant hemorrhagic risk, especially in complex airway surgery and registry-level tracheal procedure cohorts [14,15].

### 4.9. Limitations

This review has several limitations. First, the included evidence was predominantly retrospective, observational, single-center, or registry-based. No randomized trial specifically evaluating invasive procedural safety during ECMO was identified. In addition, the review protocol was not prospectively registered. This limits the possibility of independently verifying whether eligibility criteria, outcome prioritization, procedural domains, and synthesis decisions were fully predefined before completion of the review, and therefore introduces a potential risk of post hoc methodological decisions. Nevertheless, transparency was strengthened by reporting the complete search strategies, explicit eligibility criteria, standardized extraction framework, study-level outcome data, and methodological quality assessment in the Supplementary Materials. A further important limitation concerns the interpretation of procedural safety from predominantly retrospective observational evidence. Most included studies were retrospective, single-center, uncontrolled, or registry-based analyses, and several procedural domains were represented mainly by small case series. Consequently, the reported outcomes are susceptible to selection bias and confounding by indication. Patients undergoing invasive procedures during ECMO were frequently selected because clinicians considered the intervention potentially life-saving, technically feasible, or compatible with an acceptable prognosis, whereas patients considered unlikely to benefit may not have undergone the procedure. Conversely, procedures such as emergency laparotomy, thoracic surgery for bleeding or infection, pulmonary embolism intervention, and ECLS-to-LVAD transition were commonly performed in patients with severe underlying complications and advanced physiological instability. Therefore, higher bleeding or mortality rates cannot be attributed solely to the procedure itself, while favorable outcomes from selected expert-center series should not be generalized to the broader ECMO population.

Second, the included studies were heterogeneous in ECMO indication, ECMO configuration, procedural type, urgency, anticoagulation strategy, baseline illness severity, and outcome definitions. Third, bleeding definitions varied substantially across studies. Some authors reported major bleeding, whereas others reported hemothorax, transfusion, surgical-site bleeding, reoperation for bleeding, or clinically relevant hemorrhage. This heterogeneity prevented meta-analysis and required narrative synthesis. Fourth, thrombotic and circuit-related complications were inconsistently reported, and oxygenator or circuit exchange was not uniformly captured.

Fifth, several studies included mixed populations, mixed procedures, or mixed extracorporeal support modalities, including ECMO and cardiopulmonary bypass. Only ECMO-specific data were extracted when separable, but residual heterogeneity remains. Finally, many procedures were performed in high-volume referral centers, which may limit generalizability to centers without comparable ECMO, surgical, interventional, and transfusion support.

### 4.10. Future Directions

Future studies should use standardized definitions of procedure-related bleeding, major bleeding, transfusion burden, thrombotic events, circuit complications, oxygenator exchange, and reintervention. Among the procedural domains identified in this review, several may become suitable for future quantitative synthesis once additional studies with standardized outcome definitions are available. Non-elective thoracic surgery during VV-ECMO may permit pooling of major bleeding, hemothorax, transfusion requirements, repeat thoracotomy, circuit exchange, and short-term mortality outcomes. Emergency abdominal surgery and decompressive laparotomy may be evaluated using transfusion burden, reintervention for hemorrhage, restoration of ECMO flow, abdominal closure, and hospital mortality. Lung transplantation with intraoperative or perioperative ECMO/ECLS may also be appropriate for future quantitative analyses of blood loss, transfusion requirements, hemothorax, thromboembolism, reoperation, renal replacement therapy, and survival according to anticoagulation strategy or surgical approach. Finally, ECLS-to-LVAD transition studies may support pooled assessment of bleeding revision, transfusion burden, stroke, thrombosis, and early survival, particularly when surgical approach is reported consistently. Such analyses will require harmonized definitions of bleeding severity, transfusion endpoints, thrombotic and circuit events, procedural urgency, ECMO configuration, anticoagulation management, and timing of outcome assessment.

Until sufficiently standardized prospective evidence becomes available, procedural decisions during ECMO should remain individualized, multidisciplinary, and centered on the expected balance between procedural benefit, hemorrhagic risk, thrombotic risk, technical feasibility, and underlying prognosis.

## 5. Conclusions

Invasive procedures during adult ECMO may be technically achievable in selected patients and experienced centers, but safety varies markedly according to procedural domain, urgency, anticoagulation strategy, and baseline disease severity. Airway and bronchoscopic procedures, selected planned thoracic interventions, lung transplantation, cardiovascular procedures, and mechanical circulatory support-related surgery can be performed with acceptable procedural safety in carefully selected patients. Conversely, emergency abdominal surgery, abdominal exploration, decompressive laparotomy for abdominal compartment syndrome, thoracic bleeding, COVID-19-related thoracic interventions, and critical pulmonary embolism procedures are technically performable rescue interventions associated with high bleeding, transfusion, reintervention, circuit-related complication, and mortality burden.

Bleeding remains the dominant safety concern, whereas thrombotic and circuit-related complications are less consistently reported but clinically relevant. Because outcome definitions and procedural indications are heterogeneous, quantitative pooling was not appropriate. A procedure-centered, multidisciplinary approach with individualized anticoagulation management and careful planning is essential when invasive procedures are performed during ECMO. Detailed prospective, procedure-specific reporting is needed to improve risk stratification and guide clinical practice.

## Figures and Tables

**Table 1:** Characteristics of included studies.

N.	First Author, Year	Study Design/Setting	ECMO Population	Main Procedure Category	Procedure Evaluated
1	Fierro, 2019	Retrospective single-center case series	14 VV-ECMO patients, 21 procedures	Mixed non-cardiac surgery	Major non-cardiac surgery during VV-ECMO
2	Surman, 2019	Retrospective single-center series	Mixed CPB/ECMO support	Mixed non-cardiac surgery	Non-cardiac thoracic, renal, and tracheal surgery with CPB/ECMO support
3	Stokes, 2021	Retrospective cohort	8 VV-ECMO patients, 9 interventions	Airway/bronchoscopic procedures	High-risk airway interventions and whole-lung lavage
4	Meyer, 2021	Retrospective single-center series	11 patients, 14 bronchoscopies	Airway/bronchoscopic procedures	Rigid bronchoscopy and bronchotracheal stenting under ECMO
5	Koryllos, 2021	Retrospective single-center series	24 patients	Thoracic surgery	Major cardiopulmonary resections with intraoperative ECMO
6	Schweigert, 2022	Multicenter observational cohort	127 patients, 10 with ECMO	Thoracic surgery	Non-elective major lung surgery for infectious lung abscess
7	Taieb, 2019	Case-matched propensity-score study	35 ECMO and 42 non-ECMO ICU patients	Abdominal surgery	Emergency abdominal surgery during ECMO
8	Ius, 2016	Retrospective single-center cohort	595 lung transplant recipients, 170 with ECMO	Lung transplantation	Intraoperative ECMO during lung transplantation
9	Orlitová, 2023	Retrospective single-center cohort	703 lung transplantations, 156 with ECLS	Lung transplantation	Perioperative ECLS-related complications in lung transplantation
10	Ried, 2018	Retrospective registry analysis	418 VV-ECMO patients	Thoracic bleeding/surgery	Thoracic bleeding complications during VV-ECMO
11	Beyls, 2025	Retrospective bicenter propensity-matched cohort	372 VV-ECMO patients, 44 surgical	Thoracic surgery	Perioperative VV-ECMO for non-elective thoracic surgery
12	Qi, 2024	Retrospective cohort	324 lung transplant patients	Lung transplantation	Heparin-free perioperative ECMO in lung transplantation
13	Jena, 2026	Multicenter retrospective ICU cohort	56 abdominal explorations among 1386 ECMO patients	Abdominal surgery	Abdominal exploration during ECMO
14	Suzuki, 2025	ELSO Registry analysis	269 adult ECMO patients	Airway/tracheal procedures	Tracheal surgical and bronchoscopic procedures
15	Onorati, 2026	Retrospective single-center series	24 patients, 28 procedures	Airway surgery	Airway surgery and high-risk rigid bronchoscopy under VV-ECMO
16	Wang, 2022	Retrospective single-center series	13 VV-ECMO patients	Bronchoscopic biopsy	Transbronchial lung cryobiopsy during VV-ECMO
17	Almeida, 2026	Prospective database/observational series	9 COVID-19 VV-ECMO patients	Thoracic surgery	Emergency lobectomy or pneumonectomy under ECMO
18	Lubnow, 2025	Retrospective observational study	1643 ECMO patients, 47 ACS	Abdominal surgery	Decompressive laparotomy for abdominal compartment syndrome
19	Vajter, 2024	Retrospective single-center observational study	109 lung transplant patients	Lung transplantation	Intraoperative ECMO anticoagulation during lung transplantation
20	Ruszel, 2021	Retrospective single-center cohort	77 lung transplantations, 40 with support	Lung transplantation	Central/peripheral ECMO or CPB during lung transplantation
21	Zwaenepoel, 2023	Retrospective single-center study	7 COVID-19 VV-ECMO patients, 14 VATS procedures	Thoracic surgery	VATS in COVID-19 patients on VV-ECMO
22	Akil, 2023	Prospective enrollment, retrospective analysis	92 patients with low-flow VV-ECLS	Thoracic surgery	Non-intubated versus intubated VATS lung volume reduction surgery
23	Kawashima, 2025	Observational cohort	20 PAH lung transplant patients	Lung transplantation	Postoperative central VA-ECMO and delayed chest closure
24	Redivo, 2022	Retrospective medical record review	8 ECMO patients, 16 bronchoscopies	Bronchoscopic procedures	Flexible bronchoscopy during ECMO
25	Schmidt, 2019	Retrospective single-center series	16 critically ill patients, 11 on ECMO	Airway/bronchoscopic procedures	Bronchoscopic cryoextraction of airway blood clots
26	Sommerauer, 2019	Retrospective registry analysis	418 VV-ECMO patients, 29 thoracic surgery	Thoracic surgery	Non-elective thoracic surgery during VV-ECMO
27	Huang, 2021	Retrospective single-center series	22 patients	Thoracic surgery	Perioperative ECMO during thoracic surgery
28	Kim, 2021	Observational study	63 thoracic surgery patients	Thoracic surgery	High-risk thoracic surgery with ECMO
29	Laverty, 2024	Retrospective cohort	25 COVID-19 and 38 control VV-ECMO patients	Thoracic intervention	Tube thoracostomy during VV-ECMO
30	Zhang, 2022	Retrospective single-center series	15 patients	Thoracic surgery	Protective ECMO use in complex thoracic surgery
31	Spaggiari, 2021	Prospective preliminary series	6 patients	Thoracic/airway surgery	ECMO-assisted tracheal sleeve pneumonectomy
32	McCann, 2019	Retrospective observational cohort	355 ECMO patients	Abdominal surgery	Emergency laparotomy during ECMO
33	Glowka, 2018	Retrospective single-center study	175 ECMO patients, 11 DL	Abdominal surgery	Decompressive laparotomy for abdominal compartment syndrome
34	Schulz, 2020	Retrospective single-center analysis	421 ECMO patients, 8 OA	Abdominal surgery	Emergency laparotomy and open abdomen therapy
35	Amata, 2020	Retrospective observational cohort	134 VV-ECMO patients	Gastrointestinal endoscopy	Endoscopic treatment of gastrointestinal bleeding during ECMO
36	Park, 2026	Retrospective comparative cohort	136 bilateral lung transplantations	Lung transplantation	VATS versus clamshell bilateral lung transplantation with ECMO
37	Zubarevich, 2022	Retrospective single-center cohort	35 ECLS-bridged patients	Mechanical circulatory support	Durable LVAD implantation after ECLS bridge
38	Potapov, 2021	European registry, propensity-adjusted analysis	531 VA-ECLS patients	Mechanical circulatory support	Durable LVAD implantation after VA-ECLS
39	Sorensen, 2020	Retrospective single-center cohort	83 LVAD patients, 11 bridged with ECMO	Mechanical circulatory support	Minimally invasive LVAD implantation after ECMO/IABP bridge
40	Ljajikj, 2017	Retrospective comparative study	57 LVAD/ECLS patients	Mechanical circulatory support	LVAD implantation on ECLS with bivalirudin for HIT antibodies
41	Patel, 2022	Retrospective single-center series	45 patients	Cardiovascular surgery	Robot-assisted coronary artery bypass with peripheral ECMO
42	Lutz, 2023	Consecutive single-center series	5 patients	Vascular/oncologic surgery	Thoracoabdominal aortic replacement with oncologic surgery and ECMO/left-heart bypass
43	Takabayashi, 2024	Registry analysis	76 critical PE patients requiring ECMO	Pulmonary embolism intervention	Surgical, catheter, thrombolytic, or anticoagulation strategies in critical PE
44	Johannesen, 2020	Case series	3 patients	Thoracic surgery	Intraoperative ECMO in thoracic surgery
45	Boulos, 2020	Case series	9 VV-ECMO patients	Abdominal surgery	Early decompressive laparotomy for intra-abdominal hypertension
46	Heward, 2018	Retrospective single-center cohort	86 VV-ECMO patients	Thoracic intervention	Thoracic surgical interventions in ECMO services

**Table 2:** Summary of procedure-specific safety outcomes by procedural domain.

Procedural Domain	Included Studies	ECMO Population/Procedures	Main Bleeding and Transfusion Findings	Thrombotic/Circuit-Related Findings	Reintervention/Procedural Success	Mortality/Survival
Airway, bronchoscopic, and tracheobronchial procedures	7 studies	High-risk bronchoscopy, rigid bronchoscopy, bronchotracheal stenting, transbronchial cryobiopsy, tracheal procedures, cryoextraction	Single-center bronchoscopic series generally reported low severe bleeding. Wang et al. reported no severe bleeding, but moderate bleeding in 5/13 patients, controlled with balloon blockers. Suzuki et al. reported hemorrhagic complications in 26.0% and surgical-site bleeding in 13.0% in registry-level tracheal procedure data	DVT was reported in selected airway ECMO cohorts, including one cannula-associated DVT in Stokes et al. and three DVTs in Onorati et al.	Procedural success was high in selected series: all nine airway interventions completed in Stokes et al.; ECMO weaning successful in all Meyer et al. cases; diagnostic yield 100% in Wang et al.	Survival to discharge was 87.5% in Stokes et al. Thirty-day mortality was 21% in Onorati et al. Survival to discharge was 64.3% in Suzuki et al.
Thoracic surgery, chest drainage, and lung resections	15 studies	Non-elective thoracic surgery, hemothorax surgery, VATS, lung resections, LVRS, chest drainage, major cardiopulmonary resections	Thoracic bleeding was frequent and clinically relevant. Ried et al. reported relevant hemorrhage in 23.2% and thoracic bleeding in 9.6%; mortality was higher with thoracic bleeding, 52.5% vs. 32.7%, *p* = 0.013. Beyls et al. reported major bleeding 48% vs. 47%, *p* = 1.00, and hemothorax 36% vs. 2%, *p* < 0.001	Circuit changes were prominent in COVID-19 VATS patients: 10 circuit changes in 6/7 patients in Zwaenepoel et al. Venous thrombosis was 26.7% in Zhang et al.	Reintervention was common in thoracic bleeding cohorts: Ried et al. reported thoracic operation in 60% and repeat operation in 45.8%. Sommerauer et al. reported re-thoracotomy in 51.7% and overall reoperation rate 58.6%	Mortality varied widely: 25% at 30 days in Koryllos et al., 44.8% in Sommerauer et al., 55.6% in Almeida et al., and 57.1% in Zwaenepoel et al.
Abdominal surgery, gastrointestinal endoscopy, and decompressive laparotomy	8 studies	Emergency abdominal surgery, abdominal exploration, decompressive laparotomy, open abdomen therapy, GI endoscopy	Highest transfusion burden reported in emergency abdominal surgery. Taieb et al. reported transfusion in 77% vs. 40%; median RBC 13 vs. 3 units, FFP 9 vs. 0, platelets 12 vs. 0, all *p* < 0.001; ECMO independently associated with bleeding, OR 5.6, 95% CI 2.0–15.4, *p* = 0.001.	True thromboembolic occlusion was uncommon in Jena et al. Emergency oxygenator change was commonly required in McCann et al.	Reintervention for hemorrhage was 20% vs. 2%, *p* = 0.02, in Taieb et al. Schulz et al. reported median seven surgical procedures/NPWT changes and revision in 3/8. Endoscopic hemostasis achieved complete bleeding control in all active GI bleeding cases in Amata et al.	Mortality was high: ICU mortality 69% vs. 33% in Taieb et al.; in-hospital mortality 57% in Jena et al; mortality 72.7% in Glowka et al; mortality 50% in Schulz et al.
Lung transplantation and perioperative ECLS	7 studies	Intraoperative/perioperative ECMO or ECLS during lung transplantation	Hemothorax and transfusion were central outcomes. Orlitová et al. reported hemothorax in 25%. Kawashima et al. reported hemothorax evacuation in 45%. Park et al. reported lower blood loss with VATS vs. clamshell, 832 vs. 2789 mL, *p* < 0.001, and fewer transfusions.	Thromboembolism occurred in 14% in Orlitová et al. Qi et al. reported vein thrombosis in 30.2%, with no thrombosis-related deaths. Vajter et al. reported no patient- or circuit-related thrombotic complications with lower heparin dosing.	Revision for hemothorax was reduced with lower UFH dosing in Vajter et al. Sternal wound complications and airway interventions occurred only in the clamshell group in Park et al.	Ius et al. reported 1-year survival 93%, 83%, and 82% and 4-year survival 73%, 68%, and 69%, *p* = 0.11. Orlitová et al. reported 30-day mortality 6%. Kawashima et al. reported 90-day and 1-year survival 100%.
Cardiovascular, vascular, pulmonary embolism, and MCS-related procedures	7 studies	LVAD implantation after ECLS, LVAD on ECLS with bivalirudin, robot-assisted CABG, thoracoabdominal aortic replacement, critical PE interventions	Potapov et al. reported fewer blood products with less invasive LVAD implantation, median 12 vs. 16 units, *p* = 0.033, and lower revision for bleeding, 15.4% vs. 35.5%, *p* = 0.016. Takabayashi et al. reported major bleeding at 30 days in 54.0%, with procedure-site/surgery-related bleeding in 22.4%.	Stroke was 0% vs. 7.4% at 30 days in less invasive vs. full sternotomy LVAD implantation in Potapov et al. Patel et al. reported no stroke, myocardial infarction, or access-vessel complications up to 30 days.	Lutz et al. reported surgical revision in all five patients after thoracoabdominal aortic replacement. Ljajikj et al. reported early re-exploration for bleeding/tamponade 19% vs. 16.7%, OR 1.18, 95% CI 0.29–4.76, *p* = 0.820.	Zubarevich et al. reported in-hospital, 30-day, 6-month, and 1-year survival of 65.6%, 75.9%, 69.2%, and 62.7%. Takabayashi et al. reported 30-day mortality 30.3%. Patel et al. reported 30-day mortality 2.2%.
Mixed non-cardiac surgical procedures	2 studies	Major non-cardiac surgery during VV-ECMO or mixed CPB/ECMO support	Fierro et al. reported RBC transfusion in 52.4%, FFP in 23.8%, and platelets in 28.6% of procedures, with stable perioperative hemoglobin. Surman et al. reported severe hemorrhage in 3/12 mixed CPB/ECMO cases.	Surman et al. reported no direct CPB/ECMO-related complications.	Fierro et al. reported SpO_2_ < 90% in 50%, SpO_2_ < 80% in 15%, and vasopressor use in 66.7% of procedures.	Fierro et al. reported 1-year survival after ECMO cannulation of 50%. Mortality was not separately reported for ECMO-only cases in Surman et al.

This table provides a domain-level synthesis of the included studies. Author-year entries are used as study identifiers only. The complete numbered reference list for the included studies is provided in Table 1 and in the References section.

**Table 3:** Procedural settings associated with the greatest reported safety concerns during adult ECMO support.

Procedural Setting and Phenotype	Main Reported Safety Concerns	Representative Findings from Included Studies	Clinical Interpretation
**Emergency abdominal surgery during ECMO** Procedural phenotype: Reactive procedure during ECMO-associated critical illness	Major bleeding, high transfusion burden, reintervention for hemorrhage, high ICU or in-hospital mortality	Emergency abdominal surgery was associated with substantially increased transfusion requirements and reintervention for hemorrhage compared with non-ECMO ICU controls. Abdominal exploration cohorts also reported high in-hospital mortality in patients undergoing surgery during ECMO [7,13].	Emergency abdominal surgery represents one of the most clinically demanding scenarios during ECMO and should generally be reserved for urgent source control, hemorrhage control, bowel ischemia, perforation, or other life-saving indications.
**Decompressive laparotomy for intra-abdominal hypertension or abdominal compartment syndrome** Procedural phenotype: Reactive procedure during ECMO-associated critical illness	Bleeding risk, open abdomen management, repeated surgical procedures, high mortality related to underlying severity	Decompressive laparotomy was technically performable and associated with improvement in intra-abdominal pressure, ECMO flow, ventilation, and oxygenation in selected patients; however, mortality remained high in cohorts with severe multiorgan failure [18,33,34,48].	Decompressive laparotomy may be physiologically necessary when abdominal compartment syndrome compromises venous drainage, circuit flow, respiratory mechanics, or organ perfusion, but outcomes remain strongly dependent on baseline severity.
**Thoracic bleeding, hemothorax, and non-elective thoracic surgery** Procedural phenotype: Predominantly reactive/context-dependent	Hemothorax, major bleeding, repeat thoracotomy, transfusion, circuit change, mortality	Thoracic bleeding during VV-ECMO was associated with frequent surgical revision and higher mortality. In non-elective thoracic surgery cohorts, hemothorax and major bleeding were common, although selected comparative studies did not demonstrate increased mortality compared with medical VV-ECMO [10,11,26].	Thoracic procedures performed for bleeding, infection, air leak, or emergency source control require immediate access to surgical rescue, blood products, anticoagulation reassessment, and circuit surveillance.
**Emergency pulmonary resection or VATS in critically ill patients on VV-ECMO** Procedural phenotype: Reactive procedure during ECMO-associated critical illness	Uncontrollable surgical bleeding, repeated intervention, circuit exchange, high mortality	In COVID-19-associated thoracic surgical series, emergency lobectomy, pneumonectomy, or VATS was technically achievable but associated with relevant bleeding burden, circuit interventions, and substantial mortality [17,21].	These procedures should be considered only in highly selected patients when pulmonary source control or management of life-threatening hemorrhage is otherwise not achievable.
**Complex airway, tracheal, and bronchoscopic procedures** Procedural phenotype: Predominantly planned procedural ECMO	Procedure-site bleeding, moderate airway bleeding, cannula-associated thrombosis, ECMO-related complications	Bronchoscopic interventions and airway surgery were generally feasible in experienced centers. However, registry-level evidence identified hemorrhagic complications and surgical-site bleeding as clinically relevant events associated with poorer survival [14,15,16].	ECMO may facilitate complex airway procedures by maintaining gas exchange, but these interventions cannot be considered uniformly low-risk, particularly when major airway surgery or extensive tissue manipulation is required.
**Lung transplantation with intraoperative or perioperative ECMO/ECLS** Procedural phenotype: Planned perioperative ECMO/ECLS	Hemothorax, transfusion burden, thromboembolism, reoperation, renal replacement therapy	Lung transplantation studies consistently reported bleeding- and thrombosis-related outcomes. Hemothorax, transfusion requirements, venous thrombosis and ECLS-related complications were frequent in some cohorts, whereas selected anticoagulation or minimally invasive strategies were associated with reduced bleeding burden [8,9,12,19,20,23,36].	Planned perioperative ECMO pathways may be feasible in specialized transplant programs, but anticoagulation strategy, ECMO configuration and surgical approach are central determinants of procedural safety.
**Durable LVAD implantation after ECLS bridge** Procedural phenotype: Planned transition strategy after temporary support	Re-sternotomy for bleeding, major transfusion, stroke, thrombosis, right heart failure, organ dysfunction	Studies evaluating ECLS-to-LVAD bridging reported substantial bleeding and organ-failure burden. Less invasive implantation strategies were associated with reduced transfusion requirements and fewer revisions for bleeding in observational analyses [37,38,39,40].	LVAD implantation after ECLS should be interpreted as a high-risk transition strategy; bleeding reduction may be facilitated by less invasive approaches, but existing comparative evidence remains observational.
**Interventional or surgical treatment of critical pulmonary embolism requiring ECMO** Procedural phenotype: Reactive rescue intervention/context-dependent	Major bleeding, procedure-site bleeding, surgery-related bleeding, mortality	In registry-level evidence, major bleeding was frequent and procedure-site or surgery-related bleeding represented an important complication during ECMO-supported management of critical pulmonary embolism [43].	ECMO-supported pulmonary embolism intervention represents a rescue setting in which the balance between reperfusion benefit and hemorrhagic risk must be individualized by a multidisciplinary team.

**Abbreviations**: ECMO, extracorporeal membrane oxygenation; ECLS, extracorporeal life support; ICU, intensive care unit; LVAD, left ventricular assist device; VATS, video-assisted thoracic surgery; VV-ECMO, venovenous extracorporeal membrane oxygenation.

## Data Availability

The data supporting the findings of this systematic review are available in the article and in the Supplementary Materials.

## Supplementary Materials

The following supporting information can be downloaded at: https://www.mdpi.com/article/10.3390/jcm15124792/s1. Supplementary File S1: Complete Search Strategies for PubMed/MEDLINE and Scopus; Supplementary Table S1: Full Extraction of Procedure-Specific Safety Outcomes; Supplementary Table S2: Joanna Briggs Institute Critical Appraisal of Included Studies [46]; Supplementary File S2: PRISMA 2020 Checklist [45].

## References

[1] Fierro M.A., Dunne B., Ranney D.N., Daneshmand M.A., Haney J.C., Klapper J.A., Hartwig M.G., Bonadonna D., Manning M.W., Bartz R.R. (2019). Perioperative Anesthetic and Transfusion Management of Veno-Venous Extracorporeal Membrane Oxygenation Patients Undergoing Noncardiac Surgery: A Case Series of 21 Procedures. J. Cardiothorac. Vasc. Anesth..

[2] Surman T.L., Worthington M.G., Nadal J.M. (2019). Cardiopulmonary Bypass in Non-Cardiac Surgery. Heart Lung Circ..

[3] Stokes J.W., Katsis J.M., Gannon W.D., Rice T.W., Lentz R.J., Rickman O.B., Avasarala S.K., Benson C., Bacchetta M., Maldonado F. (2021). Venovenous Extracorporeal Membrane Oxygenation during High-Risk Airway Interventions. Interact. Cardiovasc. Thorac. Surg..

[4] Meyer S., Dincq A.S., Pirard L., Ocak S., D’Odémont J.P., Eucher P., Rondelet B., Gruslin A., Putz L. (2021). Bronchotracheal Stenting Management by Rigid Bronchoscopy under Extracorporeal Membrane Oxygenation Support: 10 Years of Experience in a Tertiary Center. Can. Respir. J..

[5] Koryllos A., Lopez-Pastorini A., Galetin T., Defosse J., Strassmann S., Karagiannidis C., Stoelben E. (2021). Use of Extracorporeal Membrane Oxygenation for Major Cardiopulmonary Resections. Thorac. Cardiovasc. Surg..

[6] Schweigert M., Dubecz A., Giraldo Ospina C.F., Spieth P., Almeida A.B., Richter T., Witzigmann H., Stein H.J. (2022). Use of Extracorporeal Membrane Oxygenation in Non-Elective Major Thoracic Surgery for Infectious Lung Abscess. Eur. J. Cardiothorac. Surg..

[7] Taieb A., Jeune F., Lebbah S., Schmidt M., Deransy R., Vaillant J.C., Luyt C.E., Trésallet C., Combes A., Bréchot N. (2019). Emergency Abdominal Surgery Outcomes of Critically Ill Patients on Extracorporeal Membrane Oxygenation: A Case-Matched Study with a Propensity Score Analysis. World J. Surg..

[8] Ius F., Sommer W., Tudorache I., Avsar M., Siemeni T., Salman J., Molitoris U., Gras C., Juettner B., Puntigam J. (2016). Five-Year Experience with Intraoperative Extracorporeal Membrane Oxygenation in Lung Transplantation: Indications and Midterm Results. J. Heart Lung Transplant..

[9] Orlitová M., Goos W., Van Slambrouck J., Degezelle K., Vanluyten C., Vandervelde C., De Beule J., Jin X., Berkmans E., De Leyn P. (2023). Complications Related to Extracorporeal Life Support in Lung Transplantation: Single-Center Analysis. J. Thorac. Dis..

[10] Ried M., Sommerauer L., Lubnow M., Müller T., Philipp A., Lunz D., Hofmann H.S. (2018). Thoracic Bleeding Complications in Patients with Venovenous Extracorporeal Membrane Oxygenation. Ann. Thorac. Surg..

[11] Beyls C., Soulier-Zaninka Q., Georges O., Nguyen M., Dheilly T., Nicolas M., Huette P., De Dominicis F., Abou-Arab O., Guinot P.G. (2025). Perioperative Veno-Venous Extracorporeal Membrane Oxygenation in Non-Elective Thoracic Surgery: A Propensity-Matched Bicentric Comparison with Medical Indication. Interdiscip. Cardiovasc. Thorac. Surg..

[12] Qi Z., Gu S., Yu X., Zhang Z., Cui X., Li C., Li M., Zhan Q. (2024). The Impact of Early Perioperative Heparin-Free Anticoagulation for Extracorporeal Membrane Oxygenation on Bleeding and Thrombotic Events in Lung Transplantation: A Retrospective Cohort Study. Ther. Adv. Respir. Dis..

[13] Jena A., Banker H., Tawk R., Perez A.N., Bhattacharjee S., Sarangi S., Seelhammer T.G., Bohman K.J., Patel B., Chaudhary S. (2026). Outcomes of Abdominal Exploration in ECMO-Supported Patients: A Multicenter ICU Cohort Study. J. Cardiothorac. Vasc. Anesth..

[14] Suzuki Y., Christie I.G., Chan E.G., Ryan J., Schuchert M.J., Murray H.N., Furukawa M., Sanchez P.G. (2025). Extracorporeal Membranous Oxygenation Associated with Tracheal Procedures: An Extracorporeal Life Support Organization Registry Analysis. ASAIO J..

[15] Onorati I., Radu D.M., Maiolino E., Suriano I., Juvin C., Peretti M., Portela A.M., Bardet J., Venissac N., Mouhamed M. (2026). The Role of Extracorporeal Membrane Oxygenation in Airway Surgery: Experience from a French Referral Centre for Tracheobronchial Diseases. Eur. J. Cardiothorac. Surg..

[16] Wang S., Zhou G., Feng Y., Zhang Y., Tian Y., Gu S., Wu X., Li M., Wang D., Li Y. (2022). Feasibility of Transbronchial Lung Cryobiopsy in Patients with Veno-Venous Extracorporeal Membrane Oxygenation Support. ERJ Open Res..

[17] Almeida A.B., Schweigert M., Spieth P., Dubecz A., de Abreu M.G., Richter T., Kellner P. (2026). Outcome of Emergency Pulmonary Lobectomy under ECMO Support in Patients with COVID-19. Thorac. Cardiovasc. Surg..

[18] Lubnow M., Koch C.T., Malfertheiner M.V., Foltan M., Philipp A., Lunz D., Schlitt H.J., Brennfleck F., Dietl B., Hamer O.W. (2025). Prevalence, Predictors and Decompressive Laparotomy in Abdominal Compartment Syndrome in Patients Requiring Extracorporeal Membrane Oxygenation. J. Clin. Med..

[19] Vajter J., Holubova G., Novysedlak R., Svorcova M., Vachtenheim J., Vymazal T., Lischke R. (2024). Anaesthesiologic Considerations for Intraoperative ECMO Anticoagulation during Lung Transplantation: A Single-Centre, Retrospective, Observational Study. Transpl. Int..

[20] Ruszel N., Kiełbowski K., Piotrowska M., Kubisa M., Grodzki T., Wójcik J., Kubisa B. (2021). Central, Peripheral ECMO or CPB? Comparison between Circulatory Support Methods Used during Lung Transplantation. J. Cardiothorac. Surg..

[21] Zwaenepoel B., Vandewiele K., Peperstraete H., De Ryck F., Vanpeteghem C., Malfait T., Herck I., Vandenberghe W., Van Laethem L., Defreyne L. (2023). Video-Assisted Thoracic Surgery in Critically Ill COVID-19 Patients on Venovenous Extracorporeal Membrane Oxygenation. Perfusion.

[22] Akil A., Rehers S., Ziegeler S., Ernst E., Haselmann J., Dickgreber N.J., Fischer S. (2023). Nonintubated versus Intubated Lung Volume Reduction Surgery in Patients with End-Stage Lung Emphysema and Hypercapnia. J. Clin. Med..

[23] Kawashima M., Ijiri N., Konoeda C., Toyokawa G., Nakao K., Cong Y., Hino H., Kurosawa H., Kashiwa K., Ushio M. (2025). Extended Use of Central Veno-Arterial Extracorporeal Membrane Oxygenation in Lung Transplantation for Patients with Pulmonary Arterial Hypertension. Eur. J. Cardiothorac. Surg..

[24] Redivo C.F., Lima E., Ferreira A.P., Scordamaglio P.R., Campos S.V., Ho Y.L., Rodrigues A.J. (2022). Flexible Bronchoscopy in Patients in Supportive Therapy with Oxygenation by Extracorporeal Membrane. Einstein.

[25] Schmidt L.H., Schulze A.B., Goerlich D., Schliemann C., Kessler T., Rottmann V., den Toom D., Rosenow F., Sackarnd J., Evers G. (2019). Blood Clot Removal by Cryoextraction in Critically Ill Patients with Pulmonary Hemorrhage. J. Thorac. Dis..

[26] Sommerauer L., Philipp A., Lubnow M., Müller T., Lunz D., Hofmann H.S., Ried M. (2019). Non-Elective Thoracic Surgery in Patients with Respiratory Insufficiency during Support with Veno-Venous Extracorporeal Membrane Oxygenation. Zentralbl. Chir..

[27] Huang W., Ye H., Cheng Z., Liao X., Wang L., Li B., Liang Y., Jiang H. (2021). Outcomes from the Use of Perioperative Extracorporeal Membrane Oxygenation in Patients Undergoing Thoracic Surgery: An 8-Year Single-Center Experience. Med. Sci. Monit..

[28] Kim D.H., Park J.M., Son J., Lee S.K. (2021). Multivariate Analysis of Risk Factor for Mortality and Feasibility of Extracorporeal Membrane Oxygenation in High-Risk Thoracic Surgery. Ann. Thorac. Cardiovasc. Surg..

[29] Laverty R.B., Ivins-O’Keefe K.M., Adams A.M., Flatley M.J., Sobieszczyk M.J., Mason P.E., Sams V.G. (2024). Tube Thoracostomy Complications in Patients with ARDS Requiring ECMO: Worse in COVID-19 Patients?. Mil. Med..

[30] Zhang Y., Luo M., Wang B., Qin Z., Zhou R. (2022). Perioperative, Protective Use of Extracorporeal Membrane Oxygenation in Complex Thoracic Surgery. Perfusion.

[31] Spaggiari L., Sedda G., Petrella F., Venturino M., Rossi F., Guarize J., Galetta D., Casiraghi M., Iacono G.L., Bertolaccini L. (2021). Preliminary Results of Extracorporeal Membrane Oxygenation Assisted Tracheal Sleeve Pneumonectomy for Cancer. Thorac. Cardiovasc. Surg..

[32] McCann C., Adams K., Schizas A., George M., Barrett N.A., Wyncoll D.L.A., Camporota L. (2019). Outcomes of Emergency Laparotomy in Patients on Extracorporeal Membrane Oxygenation for Severe Respiratory Failure: A Retrospective, Observational Cohort Study. J. Crit. Care.

[33] Glowka T.R., Schewe J.C., Muenster S., Putensen C., Kalff J.C., Pantelis D. (2018). Decompressive Laparotomy for the Treatment of the Abdominal Compartment Syndrome during Extracorporeal Membrane Oxygenation Support. J. Crit. Care.

[34] Schulz S.A., Schaefer S., Richards D.C., Karagiannidis C., Thomaidis P., Heiss M.M., Bulian D.R. (2020). The Need for Emergency Laparotomy with Open Abdomen Therapy in the Course of ECMO—A Retrospective Analysis of Course and Outcome. Front. Surg..

[35] Amata M., Martucci G., Granata A., Tuzzolino F., Panarello G., Bianco C., Lorusso R., Traina M., Arcadipane A. (2020). The Role of Endoscopy as Non-Invasive Procedure to Manage Gastrointestinal Complications during Extracorporeal Membrane Oxygenation. Perfusion.

[36] Park J.H., Park S., Bae S.Y., Kim D.H., Yun T., Na B., Na K.J., Lee H.J., Park I.K., Kang C.H. (2026). Safety and Feasibility of Bilateral Lung Transplantation with Video-Assisted Thoracic Surgery. Surg. Endosc..

[37] Zubarevich A., Zhigalov K., Szczechowicz M., Arjomandi Rad A., Vardanyan R., Torabi S., Papathanasiou M., Luedike P., Koch A., Pizanis N. (2022). Rescue Extracorporeal Life Support as a Bridge to Durable Left Ventricular Assist Device. Int. J. Artif. Organs.

[38] Potapov E., Loforte A., Pappalardo F., Morshuis M., Schibilsky D., Zimpfer D., Lewin D., Riebandt J., Von Aspern K., Stein J. (2021). Impact of a Surgical Approach for Implantation of Durable Left Ventricular Assist Devices in Patients on Extracorporeal Life Support. J. Card. Surg..

[39] Sorensen E.N., Griffith B.P., Feller E.D., Kaczorowski D.J. (2020). Outcomes with Temporary Mechanical Circulatory Support before Minimally Invasive Centrifugal Left Ventricular Assist Device. J. Card. Surg..

[40] Ljajikj E., Zittermann A., Morshuis M., Börgermann J., Ruiz-Cano M., Schoenbrodt M., Gummert J., Koster A. (2017). Bivalirudin Anticoagulation for Left Ventricular Assist Device Implantation on an Extracorporeal Life Support System in Patients with Heparin-Induced Thrombocytopenia Antibodies. Interact. Cardiovasc. Thorac. Surg..

[41] Patel V., Gray Z., Alam M., Silva G.V., Simpson L., Liao K. (2022). Peripheral Extracorporeal Membrane Oxygenation Support Expands the Application of Robot-Assisted Coronary Artery Bypass. JTCVS Tech..

[42] Lutz B.M., Schaser K.D., Weitz J., Kirchberg J., Fritzsche H., Disch A.C., Busch A., Wolk S., Reeps C. (2023). Thoracoabdominal Aortic Replacement Together with Curative Oncological Surgery in Retroperitoneal and Spinal Tumours. Curr. Oncol..

[43] Takabayashi K., Yamashita Y., Morimoto T., Chatani R., Kaneda K., Nishimoto Y., Ikeda N., Kobayashi Y., Ikeda S., Kim K. (2024). Clinical Characteristics, Management Strategies and Outcomes of Critical Acute Pulmonary Embolism Requiring Extracorporeal Membrane Oxygenation: From the COMMAND VTE Registry-2. J. Intensive Care.

[44] Neri G., Mazza G., Ielapi J., Mastrangelo H., Longhini F., Bosco V., Russo A., Serapide F., Pelaia C., Bruni A. (2026). Tracheostomy during Extracorporeal Membrane Oxygenation in Adult ICU Patients: A Systematic Review. J. Clin. Med..

[45] Page M.J., McKenzie J.E., Bossuyt P.M., Boutron I., Hoffmann T.C., Mulrow C.D., Shamseer L., Tetzlaff J.M., Akl E.A., Brennan S.E. (2021). The PRISMA 2020 Statement: An Updated Guideline for Reporting Systematic Reviews. BMJ.

[46] Moola S., Munn Z., Tufanaru C., Aromataris E., Sears K., Sfetcu R., Currie M., Qureshi R., Mattis P., Lisy K., Aromataris E., Munn Z. (2020). Chapter 7: Systematic Reviews of Etiology and Risk. JBI Manual for Evidence Synthesis.

[47] Johannesen S., Deb S.J. (2020). Intraoperative Extracorporeal Membrane Oxygenation in Thoracic Surgery. Ann. Thorac. Surg..

[48] Boulos F.M., Pasrija C., DiChiacchio L., Rouse M., Raithel M., Mackowick K., Rector R., Mazzeffi M.A., Griffith B.P., Diaz J.J. (2020). Early Decompressive Laparotomy for Intra-Abdominal Hypertension Following Initiation of Venovenous Extracorporeal Membrane Oxygenation. ASAIO J..

[49] Heward E., Hashmi S.F., Malagon I., Shah R., Barker J., Rammohan K.S. (2018). The Role of Thoracic Surgery in Extracorporeal Membrane Oxygenation Services. Asian Cardiovasc. Thorac. Ann..

[50] Garofalo E., Cammarota G., Neri G., Macheda S., Biamonte E., Pasqua P., Guzzo M.L., Longhini F., Bruni A. (2022). Bivalirudin vs. Enoxaparin in Intubated COVID-19 Patients: A Pilot Multicenter Randomized Controlled Trial. J. Clin. Med..

[51] Suk P., Šrámek V., Čundrle I. (2021). Extracorporeal Membrane Oxygenation Use in Thoracic Surgery. Membranes.

[52] Rocco R. (2026). Elective Perioperative Veno-Venous Extracorporeal Membrane Oxygenation for Complex Central Airway Surgery: From Rescue Therapy to Proactive Safety Net. Eur. J. Cardiothorac. Surg..

[53] Miller T., Alberter A., Munley J., Walker P., Powell E.K. (2026). Calm before the Storm—Austere Environments—Challenging Indications (Trauma). Perfusion.

