# Supplementary material for: Safety of Invasive Procedures During Adult Extracorporeal Membrane Oxygenation: A Systematic Review
Source: J Clin Med. 2026 Jun 20;15(12):4792. doi: 10.3390/jcm15124792 (PMC13302331; doi:10.3390/jcm15124792)
Supplement: Supplementary file 1 [file jcm-15-04792-s001.zip › Supplementary Table S2.pdf]

Supplementary Table S2. Joanna Briggs Institute Critical Appraisal of Included Studies

The methodological quality of the included studies was assessed using the Joanna Briggs Institute (JBI) critical appraisal tools appropriate to each study design. The JBI Checklist for Case Series was applied to descriptive procedural series without a formal comparator group, whereas the JBI Checklist for Cohort Studies was applied to comparative observational studies, registry-based analyses, and studies evaluating associations between procedural exposures and clinical outcomes. Studies were not excluded solely on the basis of methodological quality; rather, the appraisal was used to contextualize the reliability, applicability, and interpretability of the findings.

Procedural domains including only non-comparative case-series evidence are reported in Part A only and are not repeated in Part B.

Abbreviations: ECMO, extracorporeal membrane oxygenation; VV-ECMO, venovenous extracorporeal membrane oxygenation; VA-ECMO, venoarterial extracorporeal membrane oxygenation; DVT, deep-vein thrombosis; JBI, Joanna Briggs Institute; Y, Yes; N, No; U, Unclear; NA, Not applicable.

Part A. JBI Critical Appraisal Checklist for Case Series

JBI Case Series appraisal items:
Q1: Were there clear criteria for inclusion in the case series?
Q2: Was the condition/procedure measured in a standard, reliable way for all participants?
Q3: Were valid methods used for identification of the condition/procedure for all participants?
Q4: Did the case series have consecutive inclusion of participants?
Q5: Did the case series have complete inclusion of participants?
Q6: Was there clear reporting of the demographics of the participants?
Q7: Was there clear reporting of clinical information of the participants?
Q8: Were the outcomes or follow-up results of cases clearly reported?
Q9: Was there clear reporting of the presenting site(s)/clinic(s) demographic information?
Q10: Was statistical analysis appropriate?

A1. Airway, Bronchoscopic, and Tracheobronchial Procedures — Case Series

Study / PMID	Procedure evaluated	Q1	Q2	Q3	Q4	Q5	Q6	Q7	Q8	Q9	Q10	Main methodological concerns	Appraisal decision
Stokes et al., 2021 / PMID: 34293146	High-risk airway interventions and whole-lung lavage during VV-ECMO	Y	Y	Y	Y	Y	Y	Y	Y	Y	Y	Retrospective single-center series with a very small sample size; absence of a control group; highly	Included; findings interpreted primarily as evidence of feasibility in selected

												selected patients undergoing planned VV-ECMO-supported complex airway interventions.	patients and experienced centers.
Meyer et al., 2021 / PMID: 33791047	Rigid bronchoscopy and bronchotracheal stenting under ECMO support	Y	Y	Y	U	Y	Y	Y	Y	Y	Y	Retrospective single-center experience; limited number of patients and procedures; mixed VV- and VA-ECMO configurations; no non-ECMO comparator group.	Included; procedural success and safety outcomes interpreted descriptively.
Onorati et al., 2026 / PMID: 41666305	Airway surgery and high-risk rigid bronchoscopy under planned VV-ECMO	Y	Y	Y	Y	Y	Y	Y	Y	Y	Y	Retrospective referral-center series without a comparator group; heterogeneous airway procedures; relevant potential for selection bias because patients were selected for planned VV-ECMO support.	Included; contemporary and clinically relevant series, interpreted with caution because of selection bias and lack of comparison.
Wang et al., 2022 / PMID:	Transbronchial lung cryobiopsy	Y	Y	Y	Y	Y	Y	Y	Y	Y	Y	Retrospective series of only 13 patients;	Included; supports feasibility under

36545213	during VV-ECMO											procedure performed under standardized bleeding-prevention conditions; patients unsuitable for biopsy because of uncontrolled bleeding risk may not have been represented.	carefully controlled procedural conditions.
Redivo et al., 2022 / PMID: 35649052	Flexible bronchoscopy during ECMO support	Y	Y	Y	U	U	Y	Y	Y	Y	Y	Retrospective medical-record review; only eight patients and 16 bronchoscopies; consecutive and complete inclusion of all eligible procedures not clearly documented.	Included; descriptive evidence with limited external validity.
Schmidt et al., 2019 / PMID: 31737317	Bronchoscopic cryoextraction of airway blood clots in critically ill patients, including ECMO-supported patients	Y	Y	Y	U	U	Y	Y	Y	Y	Y	Retrospective series with a mixed critically ill population; only 11 of 16 patients were supported with ECMO; some outcomes were reported for	Included; interpretation restricted to ECMO-specific findings when separable.

												the entire cohort and were not fully separable for ECMO patients.	

A2. Thoracic Surgery, Chest Drainage, and Lung Resections — Case Series

Studies evaluated in this subsection:

1. Koryllos et al., 2021 / PMID: 32268398
2. Almeida et al., 2026 / PMID: 37399834
3. Zwaenepoel et al., 2023 / PMID: 35969115
4. Zhang et al., 2022 / PMID: 33908283
5. Spaggiari et al., 2021 / PMID: 32814351

A2.1. Item-by-item JBI appraisal

JBI item	Koryllos et al., 2021	Almeida et al., 2026	Zwaenepoel et al., 2023	Zhang et al., 2022	Spaggiari et al., 2021
Q1. Clear inclusion criteria	Y	Y	Y	U	Y
Q2. Procedure measured in a standard and reliable way	Y	Y	Y	Y	Y
Q3. Valid methods for identification of the procedure/condition	Y	Y	Y	Y	Y
Q4. Consecutive inclusion of participants	U	Y	Y	U	U
Q5. Complete inclusion of participants	U	Y	Y	U	Y
Q6. Clear reporting of participant demographics	Y	Y	Y	Y	Y
Q7. Clear reporting of clinical information	Y	Y	Y	Y	Y
Q8. Clear reporting of outcomes/follow-up	Y	Y	Y	Y	Y
Q9. Clear reporting of site/setting	Y	Y	Y	Y	Y
Q10. Appropriate statistical analysis	Y	Y	Y	Y	Y

Abbreviations: JBI, Joanna Briggs Institute; Y, Yes; U, Unclear.

A2.2. Methodological considerations and appraisal decisions

Study / PMID	Procedure evaluated	Main methodological concerns	Appraisal decision
Koryllos et al., 2021 / PMID: 32268398	Major cardiopulmonary resections with intraoperative ECMO, including carinal, descending aortic, and left atrial resections	Retrospective single-center analysis based on a prospectively maintained database; heterogeneous complex resections and ECMO configurations; no comparator group; consecutive and complete inclusion of all eligible procedures not explicitly documented.	Included; useful feasibility evidence for highly selected major thoracic resections performed under intraoperative ECMO, but conclusions remain descriptive.
Almeida et al., 2026 / PMID: 37399834	Emergency lobectomy or pneumonectomy under VV-ECMO in patients with COVID-19	Retrospective analysis of a prospectively collected single-center database; very small sample size; all procedures performed in a highly selected population with severe COVID-19, localized infectious or hemorrhagic complications, and failure of non-operative treatment; no control group.	Included; provides relevant evidence for emergency pulmonary source-control surgery under VV-ECMO, but outcomes cannot be generalized beyond similarly selected patients.
Zwaenepoel et al., 2023 / PMID: 35969115	VATS procedures in critically ill COVID-19 patients supported with VV-ECMO	Retrospective single-center study with only seven surgical patients; procedures mainly undertaken for hemothorax in profoundly ill patients; high risk of confounding by indication; absence of a comparator group undergoing similar surgery without ECMO.	Included; clinically relevant descriptive evidence of bleeding and circuit burden in emergency VATS during VV-ECMO.
Zhang et al., 2022 / PMID: 33908283	Perioperative protective ECMO use during complex thoracic surgery	Retrospective single-center case series; heterogeneous surgical indications, including planned and emergency procedures; mixed VA- and VV-ECMO support; criteria for consecutive and complete inclusion were not fully explicit.	Included; supports technical feasibility, while complication and survival findings should be interpreted cautiously because of clinical heterogeneity and selection bias.
Spaggiari et al., 2021 / PMID: 32814351	ECMO-assisted tracheal sleeve pneumonectomy for cancer	Prospective preliminary single-center series with only six eligible patients, five of whom received ECMO; very small oncologically selected population; no comparator; early results only.	Included; informative preliminary feasibility evidence for a highly specialized thoracic/airway oncologic procedure under ECMO support.

A2.3. Interpretative note

The case-series evidence on thoracic surgery and lung resections during ECMO was characterized by small sample sizes, single-center expertise, heterogeneous procedural indications, and limited availability of comparator groups. Nevertheless, procedures and immediate safety outcomes were generally clearly reported. Koryllos et al. and Zhang et al. described feasibility of planned or protective ECMO support during complex thoracic resections, whereas Almeida et al. and Zwaenepoel et al. evaluated emergency thoracic interventions in severely ill COVID-19 patients, in whom mortality, bleeding, and circuit-related events were strongly influenced by the underlying critical illness and urgency of surgery. Spaggiari et al. provided prospective preliminary evidence for ECMO-assisted tracheal sleeve pneumonectomy in a very small and highly selected oncologic population. Overall, these studies support procedural feasibility in specialized centers but remain vulnerable to selection bias, confounding by indication, and limited generalizability.

A2.4. Additional Appraisal: Johannesen et al., 2020

Study: Johannesen et al., 2020
PMID: 32142811
Procedure evaluated: Intraoperative ECMO support during thoracic surgery
Study design: Case series
Population: Three patients undergoing thoracic surgical procedures with intraoperative ECMO support

JBI item	Critical appraisal question	Judgment	Rationale
Q1	Were there clear criteria for inclusion in the case series?	U	The study describes three ECMO-supported thoracic surgical cases, but formal eligibility criteria for inclusion in the series were not clearly established.
Q2	Was the condition/procedure measured in a standard and reliable way for all participants?	Y	Thoracic surgery performed with intraoperative ECMO support was clearly identifiable in all reported patients.
Q3	Were valid methods used for identification of the condition/procedure for all participants?	Y	The procedural exposure and ECMO use were directly documented in the perioperative setting.
Q4	Did the case series have consecutive inclusion of participants?	U	Consecutive inclusion of all eligible cases during the study period was not clearly stated.
Q5	Did the case series have complete inclusion of participants?	U	It is unclear whether the three reported cases represented all eligible ECMO-supported thoracic procedures in the relevant period.
Q6	Was there clear reporting of the demographics of the participants?	Y	Patient-level clinical information was sufficiently reported for interpretation of the procedural context.
Q7	Was there clear reporting of clinical information of the participants?	Y	The thoracic surgical setting and use of intraoperative ECMO were described.
Q8	Were the outcomes or follow-up results of cases clearly reported?	Y	The study reported procedural completion, ECMO decannulation after

			surgery and absence of observed ECMO-related complications or procedure-related mortality.
Q9	Was there clear reporting of the presenting site(s)/clinic(s) demographic information?	Y	The perioperative thoracic surgical setting was identifiable.
Q10	Was statistical analysis appropriate?	Y	Descriptive reporting was appropriate for a three-patient case series.

Main methodological concerns:
This was a very small, descriptive case series involving only three patients. Formal inclusion criteria, consecutive case ascertainment and completeness of inclusion were not clearly documented. The absence of a comparator group and the highly selected procedural context substantially limit external validity and prevent comparative safety conclusions.

Appraisal decision:
Included. The study contributes limited but relevant descriptive evidence regarding the technical feasibility of intraoperative ECMO support during selected thoracic surgical procedures. Its findings should be interpreted cautiously because of the extremely small sample size and absence of comparative data.

Interpretative note:
Johannesen et al. reported three thoracic surgical procedures performed with intraoperative ECMO support. No ECMO-related complications were observed, all patients were decannulated after surgery, and no procedure-related mortality was reported. Although these observations support feasibility, the very small series provides only low-volume descriptive evidence and should not be used to infer general procedural safety.

A3. Abdominal Surgery, Gastrointestinal Endoscopy, and Decompressive Laparotomy — Case Series

Studies evaluated in this subsection:

1. Schulz et al., 2020 / PMID: 33102513
2. Boulos et al., 2020 / PMID: 31425255

A3.1. Item-by-item JBI appraisal

JBI item	Schulz et al., 2020	Boulos et al., 2020
Q1. Clear inclusion criteria	Y	Y
Q2. Procedure measured in a standard and reliable way	Y	Y
Q3. Valid methods for identification of the procedure/condition	Y	Y
Q4. Consecutive inclusion of participants	Y	Y
Q5. Complete inclusion of participants	Y	Y
Q6. Clear reporting of participant demographics	Y	Y
Q7. Clear reporting of clinical information	Y	Y

Q8. Clear reporting of outcomes/follow-up	Y	Y
Q9. Clear reporting of site/setting	Y	Y
Q10. Appropriate statistical analysis	Y	Y

Abbreviations: ACS, abdominal compartment syndrome; ECMO, extracorporeal membrane oxygenation; IAH, intra-abdominal hypertension; JBI, Joanna Briggs Institute; NPWT, negative-pressure wound therapy; VV-ECMO, venovenous extracorporeal membrane oxygenation; Y, Yes.

A3.2. Methodological considerations and appraisal decisions

Study / PMID	Procedure evaluated	Main methodological concerns	Appraisal decision
Schulz et al., 2020 / PMID: 33102513	Emergency laparotomy with open abdomen therapy during ECMO	Retrospective single-center analysis including only eight patients requiring open abdomen therapy among 421 ECMO cases; very small procedural cohort; heterogeneous indications for surgery; absence of a comparator group specifically matched for severity and surgical indication; high likelihood of confounding by indication because open abdomen therapy was performed in critically unstable patients.	Included; provides relevant descriptive information on feasibility, repeated surgical interventions, fascial closure, bleeding-related packing, and mortality, but does not allow comparative safety conclusions.
Boulos et al., 2020 / PMID: 31425255	Early decompressive laparotomy for intra-abdominal hypertension or abdominal compartment syndrome after initiation of VV-ECMO	Retrospective single-center series of nine VV-ECMO patients; no untreated control group with comparable ACS/IAH; the decision to perform decompression was clinically driven; small sample size limits precision and generalizability. Nevertheless, indications, physiological effects, ECMO-flow changes, respiratory parameters, length of stay, abdominal closure and survival were clearly reported.	Included; provides clinically relevant feasibility and physiological response data for decompressive laparotomy in selected VV-ECMO patients with compromised circuit drainage.

A3.3. Interpretative note

The case-series evidence in the abdominal procedural domain focused on patients requiring emergency laparotomy with open abdomen therapy or decompressive laparotomy for intra-abdominal hypertension and abdominal compartment syndrome during ECMO support. Both studies clearly described the intervention and reported clinically relevant outcomes, but were limited by very small sample sizes, single-center design, absence of suitable comparator groups, and substantial confounding by indication.

Schulz et al. evaluated eight patients receiving open abdomen therapy during ECMO, reporting a median of seven surgical procedures or NPWT dressing changes, revision surgery in three patients, fascial closure in 75%, and mortality of 50%. Boulos et al. evaluated nine VV-ECMO patients undergoing decompressive laparotomy and showed significant improvement in ECMO flow, oxygenation, and pulmonary compliance after decompression, with no major surgical or bleeding complications reported and survival to discharge of 56%. These findings suggest that decompressive or open-abdomen procedures may be technically feasible and physiologically beneficial in selected patients, but they do not establish comparative procedural safety because the interventions were undertaken in patients with severe and potentially life-threatening complications.

A4. Lung Transplantation and Perioperative ECLS — Case Series

Study evaluated in this subsection:

1. Kawashima et al., 2025 / PMID: 40758468

Although reported as an observational cohort, this study was appraised using the JBI Checklist for Case Series because it evaluated a single perioperative management strategy in a selected group of patients without a formal comparator group.

A4.1. JBI appraisal of Kawashima et al., 2025

Study: Kawashima et al., 2025
PMID: 40758468
Procedure evaluated: Lung transplantation for pulmonary arterial hypertension with postoperative central VA-ECMO and delayed chest closure
Study design: Single-group observational procedural series
Population: 20 adult lung transplant patients with pulmonary arterial hypertension; postoperative central VA-ECMO and delayed chest closure used in 17 patients

JBI item	Critical appraisal question	Judgment	Rationale
Q1	Were there clear criteria for inclusion in the case series?	Yes	The study evaluated adult patients with pulmonary arterial hypertension undergoing lung transplantation with a defined perioperative extracorporeal support strategy.
Q2	Was the condition/procedure measured in a standard and reliable way for all participants?	Yes	Lung transplantation, central VA-ECMO use, delayed chest closure, and postoperative complications were clinically identifiable and consistently reported.
Q3	Were valid methods used for identification of the condition/procedure for all participants?	Yes	The surgical procedure and postoperative ECMO strategy were directly recorded within the transplant clinical pathway.
Q4	Did the case series have consecutive inclusion of participants?	Unclear	The available reporting does not allow definite confirmation that all eligible patients during the study period were consecutively included.

Q5	Did the case series have complete inclusion of participants?	Unclear	It is not fully clear whether the reported group represented all eligible transplant patients managed with this strategy during the relevant period.
Q6	Was there clear reporting of the demographics of the participants?	Yes	The study described the transplant population and the specific indication of pulmonary arterial hypertension.
Q7	Was there clear reporting of clinical information of the participants?	Yes	The perioperative ECMO strategy, delayed closure approach, duration of support and postoperative complications were reported.
Q8	Were the outcomes or follow-up results of cases clearly reported?	Yes	Hemothorax evacuation, thrombotic complications, ICU and hospital length of stay, and survival outcomes were reported.
Q9	Was there clear reporting of the presenting site(s)/clinic(s) demographic information?	Yes	The clinical and transplant setting was clearly identifiable.
Q10	Was statistical analysis appropriate?	Yes	Descriptive analysis was appropriate for the small single-group procedural series.

Main methodological concerns:
This was a small, selected transplant series evaluating a highly specialized perioperative strategy in patients with pulmonary arterial hypertension. The absence of a formal comparator group prevents determination of whether postoperative central VA-ECMO and delayed chest closure improved or worsened safety outcomes relative to alternative approaches. The favorable survival outcomes may reflect patient selection, institutional experience, and protocolized perioperative management.

Appraisal decision:
Included. The study provides clinically relevant descriptive evidence regarding postoperative central VA-ECMO and delayed chest closure after lung transplantation for pulmonary arterial hypertension, particularly with respect to hemothorax evacuation and short-term survival, but conclusions are limited to feasibility and institutional experience.

Interpretative note:
Kawashima et al. reported that hemothorax evacuation was required in 9 of 20 patients, whereas no thrombotic complications were reported. Despite this substantial bleeding-related reintervention burden, 90-day and 1-year survival were both 100%. These findings support the feasibility of extended postoperative central VA-ECMO and delayed chest closure in a specialized transplant setting, but do not establish comparative safety because of the small, non-comparative design.

Abbreviations: ECLS, extracorporeal life support; JBI, Joanna Briggs Institute; LTx, lung transplantation; PAH, pulmonary arterial hypertension; VA-ECMO, venoarterial extracorporeal membrane oxygenation.

A5. Cardiovascular, Vascular, Pulmonary Embolism, and Mechanical Circulatory Support-Related Procedures — Case Series

Studies evaluated in this subsection:

1. Zubarevich et al., 2022 / PMID: 34674570

2. Patel et al., 2022 / PMID: 36466372
3. Lutz et al., 2023 / PMID: 36975408

A5.1. Item-by-item JBI appraisal

JBI item	Zubarevich et al., 2022	Patel et al., 2022	Lutz et al., 2023
Q1. Clear inclusion criteria	Y	Y	Y
Q2. Procedure measured in a standard and reliable way	Y	Y	Y
Q3. Valid methods for identification of the procedure/condition	Y	Y	Y
Q4. Consecutive inclusion of participants	Y	U	Y
Q5. Complete inclusion of participants	Y	U	Y
Q6. Clear reporting of participant demographics	Y	Y	Y
Q7. Clear reporting of clinical information	Y	Y	Y
Q8. Clear reporting of outcomes/follow-up	Y	Y	Y
Q9. Clear reporting of site/setting	Y	Y	Y
Q10. Appropriate statistical analysis	Y	Y	Y

Abbreviations: CABG, coronary artery bypass grafting; CS, cardiogenic shock; ECLS, extracorporeal life support; ECMO, extracorporeal membrane oxygenation; JBI, Joanna Briggs Institute; LVAD, left ventricular assist device; Y, Yes; U, Unclear.

A5.2. Methodological considerations and appraisal decisions

Study / PMID	Procedure evaluated	Main methodological concerns	Appraisal decision
Zubarevich et al., 2022 / PMID: 34674570	Rescue ECLS as a bridge to durable LVAD implantation in patients with acute cardiogenic shock	Retrospective single-center analysis of prospectively collected data including 35 patients, all presenting with INTERMACS profile I cardiogenic shock; no comparator group; high baseline severity and clinically driven selection for LVAD implantation; adverse events after LVAD may reflect both the procedural strategy and the critical preoperative condition.	Included; provides relevant descriptive evidence on bleeding, organ failure, thrombosis and survival after durable LVAD implantation following ECLS bridge, but conclusions are limited to feasibility in selected critically ill patients.
Patel et al., 2022 / PMID: 36466372	Robot-assisted coronary artery bypass with non-emergency intraoperative peripheral ECMO support	Retrospective single-center procedural series of 45 high-risk patients; absence of a non-ECMO or alternative-support comparator; selection for planned peripheral ECMO was based on clinical and technical considerations; uncommon adverse events and	Included; supports technical feasibility and short-term procedural safety of robot-assisted CABG with planned ECMO support in carefully selected patients.

		limited sample size restrict precision.	
Lutz et al., 2023 / PMID: 36975408	Thoracoabdominal aortic replacement combined with curative oncological surgery using ECMO as partial left-heart bypass	Retrospective single-center series of only five highly selected oncological patients; no comparator group; extensive multi-stage surgery and multivisceral resection make it difficult to attribute complications specifically to ECMO-supported vascular reconstruction; high risk of selection bias and limited generalizability.	Included; provides unique descriptive evidence on technically complex oncovascular surgery with ECMO support, interpreted as highly selected feasibility evidence rather than comparative safety evidence.

A5.3. Interpretative note

The case-series evidence within the cardiovascular, vascular and mechanical circulatory support-related domain was derived from highly selected procedural populations. Zubarevich et al. described 35 patients with INTERMACS profile I cardiogenic shock bridged with ECLS to durable LVAD implantation. The study clearly reported clinically relevant adverse events, including re-sternotomy for bleeding in 42.9%, acute kidney injury requiring dialysis in 68.6%, respiratory failure in 77.1%, early LVAD thrombosis in 2.9%, and 1-year survival of 62.7%. However, the absence of a comparator group and the very high baseline severity limit attribution of these outcomes to the implantation strategy itself.

Patel et al. evaluated planned peripheral ECMO support during robot-assisted coronary artery bypass in 45 high-risk patients. Short-term outcomes were favorable, with no reported stroke, myocardial infarction or access-vessel complications up to 30 days and a 30-day mortality of 2.2%. Nevertheless, the findings reflect careful procedural selection in a specialized setting and cannot be generalized to emergency or unstable cardiac surgical populations.

Lutz et al. evaluated five consecutive patients undergoing thoracoabdominal aortic replacement and curative oncological surgery with ECMO used as partial left-heart bypass. Technical success was achieved in all cases, but every patient required surgical revision, including for hematoma, bleeding, bypass revision, liquor leak or biliary leak. Because these procedures involved extensive vascular reconstruction and multivisceral or spinal tumour resection, complications cannot be attributed specifically to ECMO support. Overall, these studies support feasibility in highly specialized clinical pathways but provide limited comparative evidence on procedural safety.

A6. Mixed Non-Cardiac Surgical Procedures — Case Series

Studies evaluated in this subsection:

1. Fierro et al., 2019 / PMID: 30795968
2. Surman et al., 2019 / PMID: 29753653

A6.1. Item-by-item JBI appraisal

JBI item	Fierro et al., 2019	Surman et al., 2019

Q1. Clear inclusion criteria	Y	Y
Q2. Procedure measured in a standard and reliable way	Y	Y
Q3. Valid methods for identification of the procedure/condition	Y	Y
Q4. Consecutive inclusion of participants	Y	Y
Q5. Complete inclusion of participants	Y	U
Q6. Clear reporting of participant demographics	Y	Y
Q7. Clear reporting of clinical information	Y	Y
Q8. Clear reporting of outcomes/follow-up	Y	Y
Q9. Clear reporting of site/setting	Y	Y
Q10. Appropriate statistical analysis	Y	Y

Abbreviations: CPB, cardiopulmonary bypass; ECMO, extracorporeal membrane oxygenation; ICU, intensive care unit; JBI, Joanna Briggs Institute; VV-ECMO, venovenous extracorporeal membrane oxygenation; Y, Yes; U, Unclear.

A6.2. Methodological considerations and appraisal decisions

Study / PMID	Procedure evaluated	Main methodological concerns	Appraisal decision
Fierro et al., 2019 / PMID: 30795968	Major non-cardiac surgery during VV-ECMO	Retrospective, non-randomized, single-center observational case series including 14 patients undergoing 21 procedures. The authors identified consecutive VV-ECMO patients and excluded short procedures such as tracheostomy or percutaneous gastrostomy and lung transplantation performed on ECMO. Although perioperative transfusion, oxygenation and hemodynamic outcomes were systematically reported, the study included heterogeneous surgical procedures and lacked a comparator group. The findings may also reflect the experience of a specialized quaternary center.	Included; provides relevant descriptive evidence regarding anesthetic, hemodynamic and transfusion management of major non-cardiac procedures during VV-ECMO, interpreted as feasibility and perioperative management information rather than comparative safety evidence.
Surman et al., 2019 / PMID: 29753653	Non-cardiac thoracic, renal and tracheal surgery performed with CPB or ECMO support	Retrospective single-center series including 12 non-cardiac surgical cases, of which only three involved ECMO and nine involved CPB support or standby. Procedures and outcomes were heterogeneous, and complications and mortality were not separately extractable for all ECMO-specific cases. In addition, the overall mixed-support series included a broad clinical spectrum, including	Included; retained because ECMO-supported non-cardiac procedures were described, but only separable ECMO-specific information should contribute to the qualitative synthesis. Findings remain descriptive and should be interpreted cautiously.

		emergency, bailout and standby support, limiting direct applicability to adult ECMO procedural safety.	

A6.3. Interpretative note

The evidence for mixed non-cardiac surgical procedures during extracorporeal support was limited to two retrospective single-center case series. Fierro et al. provided the more directly applicable evidence for the present review, evaluating 21 major non-cardiac procedures performed in 14 VV-ECMO patients. Blood product administration was frequent, with red blood cells administered in 52.4% of procedures, fresh frozen plasma in 23.8%, and platelets in 28.6%. Intraoperative physiological instability was also common, including peripheral oxygen saturation below 90% in 50% of procedures and vasopressor use in 66.7%. Nevertheless, perioperative hemoglobin values remained stable and 1-year survival after ECMO cannulation was 50%.

Surman et al. described a more heterogeneous mixed-support series of non-cardiac procedures performed with CPB or ECMO. Among 12 procedures, only three involved ECMO, whereas nine involved CPB support or standby. Severe hemorrhage occurred in three cases, and prolonged ECMO support or ICU stay occurred in seven cases; however, mortality and several complication outcomes were not separately reported for ECMO-only patients. Accordingly, this study contributes contextual and feasibility information, but has limited weight in the ECMO-specific procedural safety synthesis.

Overall, the mixed non-cardiac surgery domain supports the technical feasibility of selected invasive procedures during extracorporeal support but is characterized by substantial heterogeneity, limited sample size, absence of comparator groups, and restricted ECMO-specific outcome extraction. These limitations reduce the certainty and generalizability of conclusions regarding procedural safety.

Part B. JBI Critical Appraisal Checklist for Cohort Studies

JBI Cohort Studies appraisal items:
Q1: Were the groups similar and recruited from the same population?
Q2: Were exposures/procedural classifications measured similarly to assign participants to exposed and unexposed groups?
Q3: Was the exposure/procedure measured in a valid and reliable way?
Q4: Were confounding factors identified?
Q5: Were strategies to deal with confounding factors stated?
Q6: Were participants free of the outcome at the start of the study or at the moment of exposure?
Q7: Were outcomes measured in a valid and reliable way?
Q8: Was the follow-up time reported and sufficient to allow outcomes to occur?
Q9: Was follow-up complete and, if not, were reasons for loss to follow-up described and explored?
Q10: Were strategies to address incomplete follow-up utilized?
Q11: Was appropriate statistical analysis used?

B1. Airway, Bronchoscopic, and Tracheobronchial Procedures — Cohort or Registry-Based Studies

Study: Suzuki et al., 2025
PMID: 39918107
Procedure evaluated: Tracheal surgical and bronchoscopic procedures during ECMO support
Study design: Retrospective ELSO Registry analysis
Population: 269 adult ECMO-supported patients undergoing tracheal procedures

JBI item	Critical appraisal question	Judgment	Rationale
Q1	Were the groups similar and recruited from the same population?	Unclear	Patients were identified from the same ELSO Registry population, but surgical and bronchoscopic procedure groups may have differed substantially in indication, urgency, underlying disease, and procedural complexity.
Q2	Were exposures/procedural classifications measured similarly to assign participants to exposed and unexposed groups?	Yes	Tracheal surgical and bronchoscopic procedures were categorized within the same registry-based dataset using defined procedural information.
Q3	Was the exposure/procedure measured in a valid and reliable way?	Yes	The index procedures were identifiable within a structured international ECMO registry and were directly relevant to the study objective.
Q4	Were confounding factors identified?	Unclear	Relevant clinical factors were considered in the analysis, but the extent to which all important procedural and disease-severity confounders were captured is uncertain.
Q5	Were strategies to deal with confounding factors stated?	No	The registry analysis did not provide sufficient adjustment to fully account for differences in procedural indication, urgency, baseline severity, and patient selection between procedural subgroups.
Q6	Were participants free of the outcome at the start of the study or at the moment of exposure?	Yes	Hemorrhagic complications and surgical-site bleeding were assessed as outcomes occurring in relation to ECMO-associated tracheal procedures.
Q7	Were outcomes measured in a valid and reliable way?	Yes	Hemorrhagic complications, surgical-site bleeding, and survival to discharge were clinically relevant registry-recorded outcomes.
Q8	Was the follow-up time reported and sufficient to allow outcomes to occur?	Yes	Survival to hospital discharge and procedure-associated complications were appropriate short-term outcomes for the procedural safety question.
Q9	Was follow-up complete and, if not, were reasons for loss to follow-up described and explored?	Yes	Hospital-discharge survival and in-hospital complications are routinely captured registry outcomes; no relevant

			incomplete follow-up issue was identified for the reported endpoints.
Q10	Were strategies to address incomplete follow-up utilized?	Not applicable	The main outcomes were in-hospital procedural complications and survival to discharge; incomplete longer-term follow-up was not central to the study question.
Q11	Was appropriate statistical analysis used?	Unclear	The statistical analysis permitted identification of associations between bleeding events and survival; however, residual confounding limits causal interpretation of these associations.

Main methodological concerns:
This was a retrospective registry-based analysis including heterogeneous tracheal surgical and bronchoscopic procedures. Although the study provided the largest safety dataset in this procedural domain, differences in indication, urgency, procedural complexity, baseline clinical severity, and anticoagulation management may not have been fully controlled. Voluntary registry reporting may also have affected completeness and uniformity of complication ascertainment.

Appraisal decision:
Included. The study provides an important large-scale safety signal, particularly regarding hemorrhagic complications and surgical-site bleeding, but its findings should be interpreted cautiously because of procedural heterogeneity, possible registry-related limitations, and residual confounding.

Interpretative note:
Suzuki et al. reported clinically relevant bleeding-related outcomes in adult ECMO-supported patients undergoing tracheal procedures. Hemorrhagic complications occurred in 26.0% of patients and surgical-site bleeding in 13.0%; both were associated with poorer survival. These findings strengthen the observation that complex airway and tracheal interventions during ECMO cannot be considered uniformly low risk. However, the registry design supports association rather than causation, and the reported bleeding burden should be interpreted in the context of heterogeneous patient and procedural characteristics.

Abbreviations: ECMO, extracorporeal membrane oxygenation; ELSO, Extracorporeal Life Support Organization; JBI, Joanna Briggs Institute.

Interpretative note for the airway, bronchoscopic, and tracheobronchial domain:
The methodological appraisal of studies evaluating airway, bronchoscopic, and tracheobronchial procedures showed that most evidence derived from small, retrospective, single-center case series without comparator groups. These studies generally provided clear procedural descriptions and adequately reported immediate procedural outcomes, supporting an assessment of feasibility in selected patients. However, the small sample sizes, heterogeneous procedural indications, selection of patients treated in expert referral centers, and limited ability to control for confounding restrict the generalizability of their findings. The registry-based study by Suzuki et al. provided broader safety information and identified clinically relevant hemorrhagic complications, but remained limited by procedural heterogeneity and residual confounding.

B2. Thoracic Surgery, Chest Drainage, and Lung Resections — Cohort or Comparative Studies

Studies evaluated in this subsection:

1. Schweigert et al., 2022 / PMID: 35213707
2. Ried et al., 2018 / PMID: 30193999
3. Beyls et al., 2025 / PMID: 41134639
4. Akil et al., 2023 / PMID: 37297944
5. Sommerauer et al., 2019 / PMID: 30321882
6. Huang et al., 2021 / PMID: 34725316
7. Kim et al., 2021 / PMID: 33536388
8. Laverty et al., 2024 / PMID: 38079460
9. Heward et al., 2018 / PMID: 29444601

B2.1. Item-by-item JBI appraisal: studies 1–5

JBI item	Schweigert et al., 2022	Ried et al., 2018	Beyls et al., 2025	Akil et al., 2023	Sommerauer et al., 2019
Q1. Groups similar and recruited from the same population	Y	Y	Y	U	Y
Q2. Exposure/procedural classification measured similarly across groups	Y	Y	Y	Y	Y
Q3. Exposure/procedure measured in a valid and reliable way	Y	Y	Y	Y	Y
Q4. Confounding factors identified	Y	U	Y	Y	U
Q5. Strategies to deal with confounding stated	N	N	Y	U	N
Q6. Participants free of the outcome at the start of exposure	Y	Y	Y	Y	Y
Q7. Outcomes measured in a valid and reliable way	Y	Y	Y	Y	Y
Q8. Follow-up time reported and sufficient	Y	Y	Y	Y	Y
Q9. Follow-up complete or adequately described	Y	Y	Y	Y	Y
Q10. Strategies used to address incomplete follow-up	NA	NA	Y	NA	NA
Q11. Appropriate statistical analysis	Y	U	Y	Y	Y

B2.2. Item-by-item JBI appraisal: studies 6–9

JBI item	Huang et al., 2021	Kim et al., 2021	Laverty et al., 2024	Heward et al., 2018

Q1. Groups similar and recruited from the same population	U	U	U	Y
Q2. Exposure/procedural classification measured similarly across groups	Y	Y	Y	Y
Q3. Exposure/procedure measured in a valid and reliable way	Y	Y	Y	Y
Q4. Confounding factors identified	U	Y	Y	U
Q5. Strategies to deal with confounding stated	N	Y	Y	N
Q6. Participants free of the outcome at the start of exposure	Y	Y	Y	Y
Q7. Outcomes measured in a valid and reliable way	Y	Y	Y	Y
Q8. Follow-up time reported and sufficient	Y	Y	Y	Y
Q9. Follow-up complete or adequately described	Y	U	Y	Y
Q10. Strategies used to address incomplete follow-up	NA	NA	NA	NA
Q11. Appropriate statistical analysis	U	Y	Y	U

Abbreviations: ECMO, extracorporeal membrane oxygenation; VV-ECMO, venovenous extracorporeal membrane oxygenation; ECLS, extracorporeal life support; ARDS, acute respiratory distress syndrome; JBI, Joanna Briggs Institute; Y, Yes; N, No; U, Unclear; NA, Not applicable.

B2.3. Methodological considerations and appraisal decisions

**Study / PMID**	**Procedure evaluated**	**Main methodological concerns**	**Appraisal decision**
Schweigert et al., 2022 / PMID: 35213707	Non-elective major lung surgery for infectious lung abscess, including perioperative VV-ECMO in selected patients	Retrospective analysis of a prospectively collected multicenter database; only 10 patients received ECMO; ECMO allocation was based on functional inoperability or intolerance to one-lung ventilation, introducing confounding by indication; no adjusted strategy adequately accounting for baseline severity differences between ECMO and non-ECMO patients.	Included; provides comparative feasibility information, but mortality findings should be interpreted cautiously because ECMO patients represented a highly selected and clinically different subgroup.
Ried et al., 2018 / PMID: 30193999	Thoracic bleeding complications during VV-ECMO	Retrospective single-center analysis of prospectively collected registry data including 418 consecutive VV-ECMO patients; thoracic bleeding was a complication occurring during ECMO rather than a predefined procedural	Included; important evidence on the clinical consequences of thoracic bleeding during VV-ECMO, but associations with mortality should not be interpreted as causal procedural effects.

		exposure; absence of robust adjustment for illness severity, anticoagulation status, procedure urgency, and confounding by indication.	
Beyls et al., 2025 / PMID: 41134639	Perioperative VV-ECMO for non-elective thoracic surgery compared with VV-ECMO for medical indications	Retrospective bicenter cohort; despite propensity-score matching and Cox regression, residual confounding remains possible because surgical ECMO patients differed in indication, timing, urgency, and thoracic pathology; retrospective design and exclusion of some records with missing outcomes.	Included; methodologically one of the strongest comparative studies in the thoracic domain, supporting carefully selected perioperative VV-ECMO without demonstrating definitive equivalence of procedural risk.
Akil et al., 2023 / PMID: 37297944	Non-intubated versus intubated VATS lung volume reduction surgery with low-flow VV-ECLS	Prospectively enrolled but retrospectively analyzed single-center cohort; assignment to the non-intubated approach depended on patient choice and procedural eligibility, introducing selection bias; small sample size and limited longer-term follow-up; control for confounding was incomplete.	Included; supports feasibility of VV-ECLS-assisted LVRS, particularly in selected non-intubated patients, but comparative findings require cautious interpretation.
Sommerauer et al., 2019 / PMID: 30321882	Non-elective thoracic surgery during VV-ECMO compared with VV-ECMO patients without thoracic surgery	Retrospective non-randomized single-center registry analysis; intervention group underwent surgery because of clinically important complications, especially hemothorax, abscess, or empyema; no adjustment for confounding by indication or disease severity; no long-term survival assessment.	Included; valuable description of reoperation and bleeding burden in non-elective thoracic surgery, but comparative mortality findings remain highly confounded.
Huang et al., 2021 / PMID: 34725316	Perioperative ECMO during thoracic surgery, with comparisons by timing and ECMO configuration	Retrospective single-center study of only 22 patients; heterogeneous indications and procedures; comparisons between preoperative versus postoperative ECMO and VV-versus VA-ECMO were based on small non-randomized groups; potential selection bias and insufficient control of confounding.	Included; supports feasibility of perioperative ECMO in thoracic surgery, while subgroup comparisons should be regarded as exploratory.

Kim et al., 2021 / PMID: 33536388	High-risk thoracic surgery with ECMO support and analysis of mortality-associated factors	Observational single-center cohort of heterogeneous thoracic surgical indications; multivariable analysis was performed, but the sample size was limited relative to the number and clinical complexity of potential predictors, as reflected by wide confidence intervals; residual confounding remains likely.	Included; contributes risk-factor information for ECMO-assisted thoracic surgery, particularly regarding intraoperative arrest, but estimates are imprecise and require external validation.
Laverty et al., 2024 / PMID: 38079460	Tube thoracostomy complications in COVID-19 versus non-COVID ARDS patients receiving VV-ECMO	Retrospective single-center cohort comparing clinically and temporally different populations; limited sample size; tube thoracostomy was performed according to clinical need, with potential confounding by severity of barotrauma and underlying disease; regression analysis may not fully overcome residual confounding.	Included; provides clinically useful information on chest-tube complications during VV-ECMO, but COVID-19 versus non-COVID comparisons should be interpreted cautiously.
Heward et al., 2018 / PMID: 29444601	Thoracic surgical interventions in patients receiving VV-ECMO	Retrospective single-center cohort; broad and heterogeneous intervention category including chest drains, bronchoscopies, blockers, sternotomy, and thoracotomy; no formal adjustment for the severity and indication driving intervention; procedural complications incompletely quantified by intervention type.	Included; useful service-level description of the need for thoracic surgical involvement during VV-ECMO, but limited for estimating procedure-specific comparative safety.

B2.4. Interpretative note

The cohort and comparative evidence for thoracic surgery, chest drainage, and lung resections during ECMO was methodologically heterogeneous. Several studies were based on prospectively maintained registries or databases, but analyses were generally retrospective. The most frequent methodological limitations were confounding by indication, small ECMO-supported procedural subgroups, heterogeneous surgical indications, absence of robust adjustment for baseline severity, and limited generalizability from specialized centers.

Among the included studies, Beyls et al. provided the most structured comparative approach through propensity-score matching and multivariable survival analysis. Nevertheless, even this design cannot fully eliminate residual confounding between patients receiving perioperative VV-ECMO for thoracic surgery and patients receiving VV-ECMO for medical indications. Studies

focused on thoracic bleeding, emergency surgery, or tube thoracostomy are especially vulnerable to confounding because the procedure or complication commonly reflects a more severe underlying clinical course.

Overall, these studies support the feasibility of selected thoracic interventions during ECMO, while also confirming that emergency procedures, thoracic bleeding, hemothorax, and repeated operations constitute high-risk scenarios. The available evidence should therefore be interpreted as observational safety information rather than definitive comparative evidence of procedural benefit or harm.

B3. Abdominal Surgery, Gastrointestinal Endoscopy, and Decompressive Laparotomy — Cohort or Comparative Studies

Studies evaluated in this subsection:

1. Taieb et al., 2019 / PMID: 30719558
2. Jena et al., 2026 / PMID: 41692630
3. Lubnow et al., 2025 / PMID: 39941529
4. McCann et al., 2019 / PMID: 31301640
5. Glowka et al., 2018 / PMID: 30096634
6. Amata et al., 2020 / PMID: 32156186

B3.1. Item-by-item JBI appraisal: studies 1–3

JBI item	Taieb et al., 2019	Jena et al., 2026	Lubnow et al., 2025
Q1. Groups similar and recruited from the same population	Y	Y	Y
Q2. Exposure/procedural classification measured similarly across groups	Y	Y	Y
Q3. Exposure/procedure measured in a valid and reliable way	Y	Y	Y
Q4. Confounding factors identified	Y	Y	Y
Q5. Strategies to deal with confounding stated	Y	U	U
Q6. Participants free of the outcome at the start of exposure	Y	Y	Y
Q7. Outcomes measured in a valid and reliable way	Y	Y	Y
Q8. Follow-up time reported and sufficient	Y	Y	Y
Q9. Follow-up complete or adequately described	Y	Y	Y
Q10. Strategies used to address incomplete follow-up	NA	NA	NA
Q11. Appropriate statistical analysis	Y	Y	Y

B3.2. Item-by-item JBI appraisal: studies 4–6

JBI item	McCann et al., 2019	Glowka et al., 2018	Amata et al., 2020

Q1. Groups similar and recruited from the same population	Y	Y	Y
Q2. Exposure/procedural classification measured similarly across groups	Y	Y	Y
Q3. Exposure/procedure measured in a valid and reliable way	Y	Y	Y
Q4. Confounding factors identified	U	Y	U
Q5. Strategies to deal with confounding stated	N	U	N
Q6. Participants free of the outcome at the start of exposure	Y	Y	Y
Q7. Outcomes measured in a valid and reliable way	Y	Y	Y
Q8. Follow-up time reported and sufficient	Y	Y	Y
Q9. Follow-up complete or adequately described	Y	Y	Y
Q10. Strategies used to address incomplete follow-up	NA	NA	NA
Q11. Appropriate statistical analysis	U	Y	Y

Abbreviations: ACS, abdominal compartment syndrome; ECMO, extracorporeal membrane oxygenation; GI, gastrointestinal; ICU, intensive care unit; JBI, Joanna Briggs Institute; VV-ECMO, venovenous extracorporeal membrane oxygenation; Y, Yes; N, No; U, Unclear; NA, Not applicable.

B3.3. Methodological considerations and appraisal decisions

Study / PMID	Procedure evaluated	Main methodological concerns	Appraisal decision
Taieb et al., 2019 / PMID: 30719558	Emergency abdominal surgery in critically ill patients receiving ECMO compared with non-ECMO ICU controls	Case-matched propensity-score study providing the most structured comparative analysis in this domain. Nevertheless, patients requiring ECMO represented a clinically distinct and more severely ill population, and residual confounding may persist despite matching and multivariable analysis. The need for emergency surgery itself reflects severe complications and creates relevant confounding by indication.	Included; comparatively robust evidence demonstrating a substantial association between ECMO-supported emergency abdominal surgery and bleeding/transfusion burden, while causal interpretation remains limited.
Jena et al., 2026 / PMID: 41692630	Abdominal exploration during ECMO support	Multicenter retrospective ICU cohort analyzing 56 abdominal explorations among 1386 ECMO patients; heterogeneous indications including mesenteric ischemia, intra-abdominal	Included; useful multicenter evidence regarding indications, operative findings and mortality, but limited for estimating procedure-specific bleeding

		hemorrhage, ACS and bowel perforation; comparisons were mainly between survivors and non-survivors rather than between procedural strategies; procedure-specific bleeding outcomes were not fully quantified.	risk or comparative procedural safety.
Lubnow et al., 2025 / PMID: 39941529	Decompressive laparotomy for abdominal compartment syndrome in ECMO patients	Retrospective observational cohort with 47 ACS cases among 1643 ECMO patients; patients receiving decompressive laparotomy were more severely ill than non-operated ACS patients; treatment allocation was clinically driven; specific bleeding outcomes were incompletely quantified; the small survival difference should not be interpreted as comparative effectiveness.	Included; relevant evidence on physiological effects of decompressive laparotomy, interpreted primarily as feasibility and response data in severely ill patients.
McCann et al., 2019 / PMID: 31301640	Emergency laparotomy during ECMO for severe respiratory failure	Retrospective observational cohort with a small subset undergoing laparotomy; indications for surgery and underlying severity likely differed substantially from patients not requiring laparotomy; major bleeding and oxygenator exchange were reported, but control of confounding was limited.	Included; clinically relevant evidence on emergency laparotomy and circuit-related complications, but comparative conclusions are limited by confounding by indication.
Glowka et al., 2018 / PMID: 30096634	Decompressive laparotomy for abdominal compartment syndrome during ECMO support	Retrospective single-center cohort with only 11 decompressive laparotomy patients among 175 ECMO cases; high mortality reflects severe baseline illness; multivariable analysis identified severity-related prognostic factors, but sample size was limited and the procedural effect cannot be isolated from the severity of ACS and multiorgan dysfunction.	Included; supports the clinical relevance of ACS and decompressive laparotomy during ECMO, while mortality outcomes require cautious interpretation.
Amata et al., 2020 / PMID: 32156186	Endoscopic treatment of gastrointestinal	Retrospective observational cohort with only 14 gastrointestinal bleeding events among 134 VV-ECMO	Included; provides useful evidence regarding technical effectiveness of endoscopic hemostasis in selected VV-

	bleeding during VV-ECMO	patients; endoscopy was undertaken according to clinical need; absence of a formal comparator for alternative bleeding-management strategies; limited ability to assess selection bias and unmeasured confounding.	ECMO patients with active gastrointestinal bleeding.

B3.4. Interpretative note

The methodological quality of studies addressing abdominal surgery, gastrointestinal endoscopy, and decompressive laparotomy during ECMO was limited principally by retrospective design, clinical heterogeneity, small procedural subgroups, and strong confounding by indication. Emergency abdominal surgery, abdominal exploration and decompressive laparotomy were generally performed in patients with life-threatening complications, shock, mesenteric ischemia, abdominal compartment syndrome or multiorgan failure. Consequently, the high bleeding, transfusion and mortality burdens observed in these studies cannot be attributed solely to the invasive procedure itself.

Taieb et al. provided the most informative comparative analysis in this domain through case matching and propensity-score analysis, demonstrating substantially higher transfusion requirements and reintervention for hemorrhage in ECMO-supported patients undergoing emergency abdominal surgery. However, residual confounding remains likely because ECMO patients were clinically more complex. Jena et al., McCann et al., Glowka et al. and Lubnow et al. further characterized the high-risk nature of abdominal exploration and decompressive laparotomy, but their findings mainly describe outcomes in severely ill selected populations. In contrast, Amata et al. suggested that endoscopic hemostasis for gastrointestinal bleeding may achieve effective bleeding control in selected VV-ECMO patients, although the evidence remains observational.

Overall, the abdominal procedural domain should be considered among the highest-risk settings identified in this review, particularly for emergency surgery and decompressive interventions. The available evidence supports feasibility and potential physiological benefit in selected indications but remains insufficient for definitive comparative conclusions regarding procedural safety or survival benefit.

B4. Lung Transplantation and Perioperative ECLS — Cohort or Comparative Studies

Studies evaluated in this subsection:

1. Ius et al., 2016 / PMID: 26496786
2. Orlitová et al., 2023 / PMID: 38090325
3. Qi et al., 2024 / PMID: 39161257
4. Vajter et al., 2024 / PMID: 38585623
5. Ruszel et al., 2021 / PMID: 34838067
6. Park et al., 2026 / PMID: 41840082

B4.1. Item-by-item JBI appraisal: studies 1–3

JBI item	Ius et al., 2016	Orlitová et al., 2023	Qi et al., 2024
Q1. Groups similar and recruited from the same population	U	U	U
Q2. Exposure/procedural classification measured similarly across groups	Y	Y	Y
Q3. Exposure/procedure measured in a valid and reliable way	Y	Y	Y
Q4. Confounding factors identified	Y	Y	Y
Q5. Strategies to deal with confounding stated	Y	Y	U
Q6. Participants free of the outcome at the start of exposure	Y	Y	Y
Q7. Outcomes measured in a valid and reliable way	Y	Y	Y
Q8. Follow-up time reported and sufficient	Y	Y	Y
Q9. Follow-up complete or adequately described	Y	Y	Y
Q10. Strategies used to address incomplete follow-up	NA	NA	NA
Q11. Appropriate statistical analysis	Y	Y	Y

B4.2. Item-by-item JBI appraisal: studies 4–6

JBI item	Vajter et al., 2024	Ruszel et al., 2021	Park et al., 2026
Q1. Groups similar and recruited from the same population	U	U	U
Q2. Exposure/procedural classification measured similarly across groups	Y	Y	Y
Q3. Exposure/procedure measured in a valid and reliable way	Y	Y	Y
Q4. Confounding factors identified	Y	U	Y
Q5. Strategies to deal with confounding stated	U	N	U
Q6. Participants free of the outcome at the start of exposure	Y	Y	Y
Q7. Outcomes measured in a valid and reliable way	Y	Y	Y
Q8. Follow-up time reported and sufficient	Y	Y	Y
Q9. Follow-up complete or adequately described	Y	Y	Y
Q10. Strategies used to address incomplete follow-up	NA	NA	NA
Q11. Appropriate statistical analysis	Y	Y	Y

Abbreviations: CPB, cardiopulmonary bypass; ECMO, extracorporeal membrane oxygenation; ECLS, extracorporeal life support; JBI, Joanna Briggs Institute; LTx, lung transplantation; UFH, unfractionated heparin; VATS, video-assisted thoracic surgery; Y, Yes; N, No; U, Unclear; NA, Not applicable.

B4.3. Methodological considerations and appraisal decisions

Study / PMID	Procedure evaluated	Main methodological concerns	Appraisal decision

Ius et al., 2016 / PMID: 26496786	Intraoperative ECMO during lung transplantation, comparing no ECMO, a priori ECMO and intraoperatively required ECMO groups	Large retrospective single-center cohort with clearly defined ECMO exposure groups and clinically relevant survival assessment. However, patients requiring ECMO had a higher baseline risk profile and different intraoperative trajectories than patients transplanted without ECMO. Although statistical analysis considered prognostic factors, residual confounding by indication remains likely.	Included; provides important comparative mid-term outcome data, but the absence of randomized allocation limits causal interpretation of ECMO-associated outcomes.
Orlitová et al., 2023 / PMID: 38090325	Perioperative ECLS-related complications in lung transplantation	Large retrospective single-center cohort including 703 transplantations, with 156 patients receiving perioperative ECLS. Supported patients were clinically distinct from non-supported recipients, and complications were attributable both to ECLS and to baseline transplant complexity. The observational design limits separation of support-related harm from underlying severity.	Included; valuable characterization of complication burden in perioperative ECLS, including hemothorax, thromboembolism and renal replacement therapy.
Qi et al., 2024 / PMID: 39161257	Early perioperative heparin-free ECMO strategy in lung transplantation across VV-, VA- and VV-A ECMO configurations	Retrospective cohort evaluating a standardized heparin-free approach in a large transplant population. Comparisons among ECMO configurations are potentially affected by indication and patient severity, because configuration choice was clinically driven. Vein thrombosis and bleeding outcomes were clearly reported, but absence of a heparinized control group limits conclusions on comparative safety of the anticoagulation strategy.	Included; provides clinically relevant safety data on bleeding and thrombosis under a heparin-free perioperative ECMO strategy, interpreted as observational evidence.
Vajter et al., 2024 / PMID: 38585623	Intraoperative anticoagulation strategy during central VA-ECMO for lung transplantation	Retrospective single-center observational cohort of 109 patients successfully weaned from intraoperative central VA-ECMO. Lower UFH exposure was associated with reduced blood loss, blood product use and fewer revisions for	Included; useful evidence supporting lower-heparin intraoperative strategies in selected transplant patients, but comparative findings remain vulnerable to confounding.

		hemothorax; however, anticoagulation dosing was not randomized, and relevant patient or procedural differences may have influenced treatment allocation and outcomes.	
Ruszel et al., 2021 / PMID: 34838067	Central ECMO, peripheral ECMO or CPB during lung transplantation	Retrospective single-center cohort with comparison among different intraoperative extracorporeal support methods. Support type was selected clinically rather than randomly, and groups likely differed in baseline severity, anatomical considerations and intraoperative instability. Limited adjustment for confounding restricts comparisons of complications and survival.	Included; provides descriptive comparative information across extracorporeal support strategies, interpreted cautiously because of treatment-selection bias.
Park et al., 2026 / PMID: 41840082	VATS versus clamshell bilateral lung transplantation performed with ECMO support	Retrospective comparative cohort evaluating two surgical approaches in ECMO-supported bilateral lung transplantation. Although operative time, blood loss, transfusion requirements and postoperative complications were clearly reported, selection of the surgical approach may have depended on patient characteristics, technical feasibility and temporal changes in institutional practice.	Included; clinically informative comparative evidence suggesting reduced surgical trauma with VATS, but non-randomized allocation limits definitive conclusions.

B4.4. Interpretative note

The lung transplantation and perioperative ECLS domain contained some of the largest and most clinically structured observational studies in the review. Compared with the small procedural series in other domains, these studies generally reported defined ECMO configurations, perioperative strategies, bleeding outcomes, thrombotic events and survival with greater completeness. However, all evidence remained observational, and ECMO use, ECMO configuration, anticoagulation strategy and surgical approach were selected according to clinical indications rather than randomly assigned.

The principal methodological concern across this domain was therefore confounding by indication. Patients requiring intraoperative or postoperative ECMO were frequently more critically ill, had greater procedural complexity or required specific support strategies because of underlying cardiopulmonary instability. Similarly, comparisons between heparin-free or lower-heparin

protocols, central versus peripheral ECMO, and VATS versus clamshell approaches may reflect both treatment effects and patient-selection factors.

Within these limitations, the included studies consistently identified bleeding, hemothorax, transfusion requirements, thromboembolism and renal replacement therapy as central safety outcomes. Ius et al. showed that intraoperative ECMO did not significantly worsen mid-term survival despite higher-risk patient profiles. Orlitová et al. documented a substantial ECLS-related complication burden, with hemothorax and thromboembolism among the most frequent events. Qi et al. and Vajter et al. suggested that reduced or absent perioperative heparin exposure may be feasible in selected patients, although thrombosis remained clinically relevant in the heparin-free cohort. Park et al. suggested lower blood loss and transfusion requirements with a minimally invasive VATS approach compared with clamshell transplantation.

Overall, the methodological quality of this domain was higher than that of many small case-series domains because of larger samples and comparative analyses. Nevertheless, the absence of randomized allocation, potential treatment-selection bias and residual confounding require cautious interpretation of apparent differences in safety outcomes.

B5. Cardiovascular, Vascular, Pulmonary Embolism, and Mechanical Circulatory Support-Related Procedures — Cohort or Comparative Studies

Studies evaluated in this subsection:

1. Potapov et al., 2021 / PMID: 33547707
2. Sorensen et al., 2020 / PMID: 32579786
3. Ljajikj et al., 2017 / PMID: 29049666
4. Takabayashi et al., 2024 / PMID: 39497225

B5.1. Item-by-item JBI appraisal

JBI item	Potapov et al., 2021	Sorensen et al., 2020	Ljajikj et al., 2017	Takabayashi et al., 2024
Q1. Groups similar and recruited from the same population	Y	U	Y	U
Q2. Exposure/procedural classification measured similarly across groups	Y	Y	Y	Y
Q3. Exposure/procedure measured in a valid and reliable way	Y	Y	Y	Y
Q4. Confounding factors identified	Y	Y	Y	Y
Q5. Strategies to deal with confounding stated	Y	U	Y	U
Q6. Participants free of the outcome at the start of exposure	Y	Y	Y	Y
Q7. Outcomes measured in a valid and reliable way	Y	Y	Y	Y
Q8. Follow-up time reported and sufficient	Y	Y	Y	Y
Q9. Follow-up complete or adequately described	Y	Y	Y	Y

Q10. Strategies used to address incomplete follow-up	NA	NA	NA	NA
Q11. Appropriate statistical analysis	Y	Y	Y	Y

Abbreviations: CABG, coronary artery bypass grafting; ECMO, extracorporeal membrane oxygenation; ECLS, extracorporeal life support; FS, full sternotomy; HIT, heparin-induced thrombocytopenia; JBI, Joanna Briggs Institute; LIS, less invasive surgery; LVAD, left ventricular assist device; PE, pulmonary embolism; VA-ECLS, venoarterial extracorporeal life support; Y, Yes; U, Unclear; NA, Not applicable.

B5.2. Methodological considerations and appraisal decisions

Study / PMID	Procedure evaluated	Main methodological concerns	Appraisal decision
Potapov et al., 2021 / PMID: 33547707	Durable LVAD implantation after VA-ECLS: full sternotomy versus less invasive surgical approach	European registry-based comparative study including a large population and propensity-adjusted analyses. Nevertheless, the less invasive surgery subgroup was substantially smaller than the full sternotomy group, and surgical approach was not randomly allocated. Residual confounding related to institutional preference, technical eligibility and patient stability may persist.	Included; provides comparatively robust observational evidence that less invasive LVAD implantation may reduce transfusion burden and revision for bleeding after VA-ECLS bridge.
Sorensen et al., 2020 / PMID: 32579786	Minimally invasive durable LVAD implantation after bridge with ECMO, IABP or no temporary mechanical circulatory support	Retrospective analysis of a prospectively collected single-center database; only 11 patients were bridged with ECMO and all were INTERMACS profile I, while comparator groups had substantially different baseline severity; no robust adjustment fully addressing this imbalance; procedure performed in a specialized high-volume center.	Included; provides useful comparative outcome data after minimally invasive LVAD implantation, but similar postoperative outcomes cannot be interpreted as equivalence because of small ECMO sample size and important baseline differences.
Ljajikj et al., 2017 / PMID: 29049666	LVAD implantation on ECLS using bivalirudin versus heparin in patients with HIT antibodies	Retrospective comparative study with clinically relevant treatment groups and propensity-score matching. However, the total sample size was limited, anticoagulant assignment was clinically determined rather than randomized, and	Included; provides relevant comparative evidence on anticoagulation during LVAD implantation on ECLS, showing no significant excess bleeding-related re-exploration with

		relatively uncommon bleeding and neurological events limit precision of effect estimates.	bivalirudin, while remaining subject to residual confounding.
Takabayashi et al., 2024 / PMID: 39497225	Surgical intervention, catheter intervention, systemic thrombolysis or anticoagulation-only strategies in critical acute pulmonary embolism requiring ECMO	Registry-based observational analysis of 76 patients with critical pulmonary embolism requiring ECMO. Treatment strategies were determined by clinical presentation, center practice and patient instability rather than random allocation. Groups likely differed substantially in severity, contraindications and timing of intervention; therefore, comparisons of mortality across strategies are highly susceptible to confounding by indication.	Included; important evidence regarding bleeding burden and outcomes in critical pulmonary embolism requiring ECMO, interpreted as observational safety information rather than comparative evidence favoring a specific treatment strategy.

B5.3. Interpretative note

The cohort and comparative studies in the cardiovascular, vascular, pulmonary embolism and mechanical circulatory support-related domain provided clinically relevant data on major bleeding, transfusion burden, revision for bleeding, stroke, thrombosis and survival. Potapov et al. represented the largest analytic study in this domain and suggested that less invasive LVAD implantation after VA-ECLS was associated with fewer blood products and fewer revisions for bleeding than full sternotomy. However, treatment allocation was non-randomized and residual confounding related to procedural eligibility and institutional practice cannot be excluded.

Sorensen et al. reported that patients bridged with ECMO to minimally invasive LVAD implantation required more intraoperative red-cell transfusions than patients with IABP or no temporary support, although subsequent bleeding, stroke, infection rates and survival were not significantly different. Importantly, all ECMO patients had INTERMACS profile I disease, indicating substantially greater baseline clinical severity and limiting direct comparability.

Ljajikj et al. evaluated bivalirudin versus heparin in patients undergoing LVAD implantation on ECLS in the setting of HIT antibodies. Although early surgical re-exploration for bleeding or tamponade was similar between groups, the retrospective design and small sample size mean that absence of statistically significant differences should not be interpreted as definitive equivalence.

Takabayashi et al. documented a substantial bleeding burden in patients with critical pulmonary embolism requiring ECMO, with major bleeding at 30 days in 54.0% and procedure-site or surgery-related bleeding in 22.4%. Differences in mortality among surgical, catheter-based, thrombolytic and anticoagulation-only strategies are clinically informative but remain strongly influenced by treatment-selection bias and confounding by indication.

Overall, this procedural domain included several comparative studies with greater analytic depth than simple case series, but the evidence remained observational. Apparent advantages of less

invasive surgical approaches or specific anticoagulation strategies should therefore be interpreted as hypothesis-generating findings requiring validation in prospective multicenter cohorts.
