# Supplementary material for: Safety of Invasive Procedures During Adult Extracorporeal Membrane Oxygenation: A Systematic Review
Source: J Clin Med. 2026 Jun 20;15(12):4792. doi: 10.3390/jcm15124792 (PMC13302331; doi:10.3390/jcm15124792)
Supplement: Supplementary file 1 [file jcm-15-04792-s001.zip › Supplementary_File_S1.pdf]

Supplementary File S1. Complete Search Strategies for PubMed/MEDLINE and Scopus

Manuscript title: *Safety of Invasive Procedures During Adult Extracorporeal Membrane Oxygenation: A Systematic Review*

Search Strategy and Reproducibility

A systematic literature search was conducted in PubMed/MEDLINE and Scopus to identify studies evaluating the safety of invasive procedures performed during ECMO or ECLS in adult patients. The final searches were performed in April 2026. Studies focusing on tracheostomy or tracheotomy were excluded because this procedural domain had recently been addressed in a dedicated systematic review.

Table S1.1. PubMed/MEDLINE Search Strategy

Item	Details
Database	PubMed/MEDLINE
Date of final search	April 2026
Records retrieved	234
Exclusions incorporated in the query	Tracheostomy/tracheotomy; neonatal and pediatric populations

Complete PubMed/MEDLINE Search String

```
(
  "Extracorporeal Membrane Oxygenation"[Mesh]
  OR ECMO[tiab]
  OR ECLS[tiab]
  OR "extracorporeal membrane oxygenation"[tiab]
  OR "extracorporeal life support"[tiab]
)
AND
(
  "chest tube"[tiab]
  OR "chest tubes"[tiab]
  OR thoracostom*[tiab]
  OR "tube thoracostomy"[tiab]
  OR "pleural drainage"[tiab]
  OR "pleural drain"[tiab]
  OR "pigtail catheter"[tiab]
  OR pneumothorax[tiab]
  OR hemothorax[tiab]
  OR haemothorax[tiab]
  OR "Bronchoscopy"[Mesh]
  OR bronchoscop*[tiab]
```

```
  OR "interventional bronchoscopy"[tiab]
  OR "therapeutic bronchoscopy"[tiab]
  OR "airway procedure"[tiab]
  OR "airway procedures"[tiab]
  OR thoracotomy[tiab]
  OR laparotomy[tiab]
  OR "thoracic surgery"[tiab]
  OR "abdominal surgery"[tiab]
  OR "vascular surgery"[tiab]
  OR "emergency surgery"[tiab]
  OR "noncardiac surgery"[tiab]
  OR "non-cardiac surgery"[tiab]
  OR "surgical intervention"[tiab]
  OR "surgical interventions"[tiab]
  OR "percutaneous drainage"[tiab]
  OR "percutaneous drain"[tiab]
  OR "bedside procedure"[tiab]
  OR "bedside procedures"[tiab]
  OR "invasive procedure"[tiab]
  OR "invasive procedures"[tiab]
)
AND
(
  safety[tiab]
  OR safe[tiab]
  OR complication*[tiab]
  OR "adverse event"[tiab]
  OR "adverse events"[tiab]
  OR morbidity[tiab]
  OR bleeding[tiab]
  OR hemorrhage[tiab]
  OR haemorrhage[tiab]
  OR hemorrhagic[tiab]
  OR haemorrhagic[tiab]
  OR "Hemorrhage"[Mesh]
  OR transfusion*[tiab]
  OR "Blood Transfusion"[Mesh]
  OR "blood product"[tiab]
  OR "blood products"[tiab]
  OR "red blood cell"[tiab]
  OR "red blood cells"[tiab]
```

```
  OR "packed red blood cells"[tiab]
  OR PRBC[tiab]
  OR platelet*[tiab]
  OR plasma[tiab]
  OR anticoagulation[tiab]
  OR thrombosis[tiab]
  OR thrombotic[tiab]
  OR "circuit thrombosis"[tiab]
  OR "oxygenator exchange"[tiab]
  OR reintervention[tiab]
  OR reoperation[tiab]
  OR "procedural failure"[tiab]
)
AND
(
  adult*[tiab]
  OR "Adult"[Mesh]
  OR ICU[tiab]
  OR "intensive care"[tiab]
  OR "critically ill"[tiab]
)
NOT
(
  tracheostom*[tiab]
  OR tracheotom*[tiab]
  OR "Tracheostomy"[Mesh]
  OR "Tracheotomy"[Mesh]
  OR neonat*[tiab]
  OR pediatric*[tiab]
  OR paediatric*[tiab]
)
```

Table S1.2. Scopus Search Strategy

Item	Details
Database	Scopus
Date of final search	April 2026
Records retrieved	344
Search fields	TITLE, ABS, and TITLE-ABS-KEY
Applied limits	PUBYEAR > 2015; English language
Exclusions incorporated in the query	Tracheostomy/tracheotomy; pediatric/neonatal and animal records; case reports, reviews, meta-analyses, scoping reviews, narrative reviews, and

	preprints

Complete Scopus Search String

```
(
  TITLE (
    "extracorporeal membrane oxygenation"
    OR ECMO
    OR ECLS
    OR "extracorporeal life support"
  )
  OR ABS (
    "extracorporeal membrane oxygenation"
    OR ECMO
    OR ECLS
    OR "extracorporeal life support"
  )
)
AND TITLE-ABS-KEY (
  "chest tube"
  OR "chest tubes"
  OR thoracostom*
  OR "tube thoracostomy"
  OR "pleural drainage"
  OR "pleural drain"
  OR "pleural drains"
  OR "pigtail catheter"
  OR "pigtail catheters"
  OR pneumothorax
  OR hemothorax
  OR haemothorax
  OR bronchoscop*
  OR "interventional bronchoscopy"
  OR "therapeutic bronchoscopy"
  OR "airway procedure"
  OR "airway procedures"
  OR thoracotomy
  OR laparotomy
  OR "thoracic surgery"
  OR "abdominal surgery"
  OR "vascular surgery"
  OR "emergency surgery"
```

```
  OR "noncardiac surgery"
  OR "non-cardiac surgery"
  OR "surgical intervention"
  OR "surgical interventions"
  OR "percutaneous drainage"
  OR "percutaneous drain"
  OR "percutaneous drains"
  OR "bedside procedure"
  OR "bedside procedures"
  OR "invasive procedure"
  OR "invasive procedures"
)
AND TITLE-ABS-KEY (
  safety
  OR safe
  OR complication*
  OR "adverse event"
  OR "adverse events"
  OR morbidity
  OR bleeding
  OR hemorrhage
  OR haemorrhage
  OR hemorrhagic
  OR haemorrhagic
  OR transfusion*
  OR "blood transfusion"
  OR "blood product"
  OR "blood products"
  OR "red blood cell"
  OR "red blood cells"
  OR "packed red blood cells"
  OR PRBC
  OR platelet*
  OR plasma
  OR anticoagulation
  OR thrombosis
  OR thrombotic
  OR "circuit thrombosis"
  OR "oxygenator exchange"
  OR reintervention
  OR reoperation
```

```
  OR "procedural failure"
)
AND TITLE-ABS-KEY (
  adult*
  OR ICU
  OR "intensive care"
  OR "critically ill"
  OR human
  OR humans
  OR patient
  OR patients
)
AND NOT TITLE-ABS-KEY (
  tracheostom*
  OR tracheotom*
  OR neonat*
  OR newborn*
  OR infant*
  OR child*
  OR children
  OR pediatric*
  OR paediatric*
  OR adolescent*
  OR animal
  OR animals
  OR porcine
  OR swine
  OR pig
  OR pigs
  OR ovine
  OR sheep
  OR canine
  OR dog
  OR dogs
  OR rat
  OR rats
  OR mouse
  OR mice
)
AND NOT TITLE-ABS-KEY (
  "case report"
```

```
  OR "case reports"
  OR "case series"
  OR "systematic review"
  OR "meta-analysis"
  OR metaanalysis
  OR "scoping review"
  OR "narrative review"
  OR review
  OR preprint
  OR preprints
)
AND PUBYEAR > 2015
AND (
  LIMIT-TO ( LANGUAGE, "English" )
)
```

Final Included Evidence Base

Item	Details
PubMed/MEDLINE studies included	26
Additional unique Scopus studies included	20
Total studies included in the qualitative synthesis	46
