# Supplementary material for: Safety of Invasive Procedures During Adult Extracorporeal Membrane Oxygenation: A Systematic Review
Source: J Clin Med. 2026 Jun 20;15(12):4792. doi: 10.3390/jcm15124792 (PMC13302331; doi:10.3390/jcm15124792)
Supplement: Supplementary file 1 [file jcm-15-04792-s001.zip › Supplementary_Table_S1.pdf]

Supplementary Table S1. Full extraction of procedure-specific safety outcomes.

N	Study / PMID	Procedure	N / ECMO type	Bleeding / transfusion	Thrombosis / circuit events	Reintervention / procedural success	Mortality / survival	Main statistics
1	Fierro et al. / 30795968	Major non-cardiac surgery during VV-ECMO	14 patients, 21 procedures; VV-ECMO	RBC in 52.4%, FFP in 23.8%, platelets in 28.6%; hemoglobin stable	Not specifically reported	SpO_2 <90% in 50%; SpO_2 <80% in 15%; vasopressors in 66.7%	1-year survival 50%	Descriptive statistics only
2	Surman et al. / 29753653	Non-cardiac surgery with CPB/ECMO support	12 cases; 3 ECMO, 9 CPB/standby	Severe hemorrhage in 3/12	No direct CPB/ECMO-related complications reported	Prolonged ECMO support/ICU stay in 7 cases	Not separately extractable for ECMO-only cases	Descriptive statistics only
3	Stokes et al. / 34293146	High-risk airway interventions and whole-lung lavage	8 patients, 9 interventions; VV-ECMO	No major bleeding reported	One cannula-associated DVT	All 9 interventions completed successfully	Survival to discharge 87.5%; 1-year survival 50%	Descriptive statistics only
4	Meyer et al. / 33791047	Rigid bronchoscopy and bronchotracheal stenting	11 patients, 14 bronchoscopies; VV/VA-ECMO	Anticoagulation adapted to avoid bleeding; no significant operating-site bleeding reported	Two local cannulation-related complications	ECMO weaning successful in all cases	Not clearly procedure-specific	Descriptive statistics only
5	Koryllos et al. / 32268398	Major cardiopulmonary resections	24 patients; VV/VA/VVA-ECMO	No intraoperative complications reported	No intraoperative ECMO complications reported	Complete resection in 18/24	30-day mortality 25%; median survival 12 months	Descriptive statistics only

6	Schweigert et al. / 35213707	Non-elective major lung surgery for infectious abscess	127 patients; 10 ECMO	No intraoperative ECMO-associated complications	No ECMO-associated intraoperative complications	Surgery feasible under ECMO	Mortality 1/10 ECMO vs. 16/117 non-ECMO	ECMO not associated with mortality: OR 0.70, 95% CI 0.08–5.91, p = 0.74
7	Taieb et al. / 30719558	Emergency abdominal surgery	35 ECMO vs. 42 non-ECMO ICU controls	Transfusion 77% vs. 40%; RBC 13 vs. 3 units; FFP 9 vs. 0; platelets 12 vs. 0	Not primary outcome	Reintervention for hemorrhage 20% vs. 2%	ICU mortality 69% vs. 33%; perioperative mortality 11% vs. 12%	Transfusions all p < 0.001; reintervention p = 0.02; ECMO associated with bleeding OR 5.6, 95% CI 2.0–15.4, p = 0.001
8	Ius et al. / 26496786	Intraoperative ECMO during lung transplantation	595 LTx; 170 ECMO	More postoperative complications in ECMO groups	Not specified in abstract-level extraction	Not specified	1-year survival 93%, 83%, 82%; 4-year survival 73%, 68%, 69%	Survival p = 0.11; intraoperative ECMO not risk factor for mortality
9	Orlitová et al. / 38090325	Perioperative ECLS in lung transplantation	156 ECLS among 703 LTx	Hemothorax 25%	Thromboembolism 14%; CRRT 19%	ECLS-related complication in 67%	30-day mortality 6%	Descriptive statistics only
10	Ried et al. / 30193999	Thoracic bleeding during VV-ECMO	418 VV-ECMO	Relevant hemorrhage 23.2%; thoracic bleeding 9.6%	Not primary outcome	Thoracic operation in 60%; repeat operation in 45.8%	Mortality 52.5% with thoracic bleeding vs. 32.7%	ECMO duration p = 0.035; hospital stay p = 0.002;

							without bleeding	mortality p = 0.013
11	Beyls et al. / 41134639	Perioperative VV-ECMO for non-elective thoracic surgery	372 VV-ECMO; 44 perioperative	Hemothorax 36% vs. 2%; major bleeding 48% vs. 47%	Thrombotic events 41% vs. 32%	Not separately detailed	90-day mortality 54% vs. 51%	Hemothorax p < 0.001; major bleeding p = 1.00; thrombosis p = 0.31; mortality p = 0.71; matched log-rank p = 0.95; HR 1.003, 95% CI 0.64–1.57
12	Qi et al. / 39161257	Heparin-free perioperative ECMO in lung transplantation	324 LTx; VV, VA, VV-A ECMO	VV group lowest intraoperative bleeding, drainage, transfusion	Vein thrombosis 30.2%; no thrombosis-related deaths	Not specified	Intraoperative bleeding and 24 h drainage predicted 1-year survival	Differences among ECMO modes not significant for vein thrombosis
13	Jena et al. / 41692630	Abdominal exploration during ECMO	56 explorations among 1386 ECMO patients	Included intra-abdominal hemorrhage indications; procedure-specific bleeding not fully quantified	True thromboembolic occlusion uncommon	Surgical intervention according to pathology	In-hospital mortality 57%	Non-survivors had higher APACHE II p < 0.001, SOFA p < 0.01, pre-ECMO lactate p < 0.001

14	Suzuki et al. / 39918107	Tracheal surgical and bronchoscopic procedures	269 ECMO patients	Hemorrhagic complications 26.0%; surgical-site bleeding 13.0%	Not primary outcome	Procedure subgroups analyzed	Survival to discharge 64.3%	Hemorrhagic complications associated with worse survival $p < 0.001$; surgical-site bleeding $p = 0.007$
15	Onorati et al. / 41666305	Airway surgery and rigid bronchoscopy	24 patients, 28 procedures; VV-ECMO	Major bleeding anticipated in indications; bleeding-specific rates not fully separated	ECMO-specific complications in 4 cases: DVT n = 3, vasoplegia n = 1	Decannulation in OR in 22 cases; delayed in 4; 2 died on ECMO	30-day mortality 21%	Descriptive statistics only
16	Wang et al. / 36545213	Transbronchial lung cryobiopsy during VV-ECMO	13 VV-ECMO patients	No severe bleeding; moderate bleeding 5/13 controlled with balloon blockers	No pneumothorax reported	Diagnostic yield 100%	No procedure-related death	Descriptive statistics only
17	Almeida et al. / 37399834	Emergency lobectomy/pneumonectomy in COVID-19	9 VV-ECMO patients	Massive pulmonary hemorrhage indication in 2/9	Pulmonary embolism present in 4/9 before surgery	ECMO weaning successful in 4/9	In-hospital mortality 5/9	Descriptive statistics only
18	Lubnow et al. / 39941529	Decompressive laparotomy for ACS	47 ACS among 1643 ECMO patients	DL reported with low complication rates; specific bleeding not fully quantified	Not specified	DL reduced IAP and improved ventilation	Survival DL 11% vs. non-DL 14%	SOFA higher in DL group $p = 0.048$; survival $p = 1.000$

19	Vajter et al. / 38585623	Intraoperative ECMO anticoagulation in LTx	109 central VA-ECMO LTx	Lower UFH associated with less blood loss and blood product use	No patient- or circuit-related thrombotic complications	Lower UFH may reduce revision for hemothorax	Mortality/survival evaluated	Comparative statistics reported in article; direction favors lower UFH
20	Ruszel et al. / 34838067	Central/peripheral ECMO or CPB during LTx	77 LTx; 40 supported	Bleeding among possible complications; not dominant extracted outcome	AKI and thromboembolic complications more frequent with support	Not specified	Central ECMO survival 30-day/1-/3-/5-year: 78/66/66/66%	AKI p = 0.005; thromboembolic complications p = 0.02
21	Zwaenepoel et al. / 35969115	VATS in COVID-19 patients on VV-ECMO	7 patients, 14 VATS	Two immediate perioperative deaths due to uncontrollable bleeding	10 circuit changes in 6 patients; one possibly related to VATS	All survivors required transarterial embolization	Mortality 57.1%	Descriptive statistics only
22	Akil et al. / 37297944	VATS-LVRS with low-flow VV-ECLS	92 patients	Prolonged chest tube therapy lower in non-intubated group	Not specifically reported	Conversion to general anesthesia in 1 patient	90-day mortality 3% vs. 7%	Chest tube removal p < 0.02; ICU stay p = 0.04; hospital stay p = 0.01
23	Kawashima et al. / 40758468	LTx for PAH with central VA-ECMO and delayed closure	20 patients	Hemothorax evacuation 45%	No thrombotic complications	Delayed chest closure used in 17/20	90-day and 1-year survival 100%	Descriptive statistics only
24	Redivo et al. / 35649052	Flexible bronchoscopy during ECMO	8 patients, 16 bronchoscopies	Moderate bleeding after biopsy in 1 procedure	No hemodynamic complications; no	Transient desaturation in 1 procedure	Not procedure-related mortality	Descriptive statistics only

				, controlled endoscopically	radiologic worsening			
25	Schmidt et al. / 31737317	Bronchoscopic cryoextraction of airway blood clots	16 patients; 11 ECMO	No severe procedure-related complications	Not specified	Successful airway recanalization in all cases; repeated in 7 patients	Not procedure-specific	Descriptive statistics only
26	Sommerauer et al. / 30321882	Non-elective thoracic surgery during VV-ECMO	29 thoracic surgery patients among 418 VV-ECMO	Hemothorax most frequent indication, 44.8%	Not specified	Re-thoracotomy in 51.7%; >2 surgeries in 31.0%; reoperation rate 58.6%	In-hospital mortality 44.8% vs. 35.7% without thoracic surgery	Mortality p = 0.326
27	Huang et al. / 34725316	Perioperative ECMO in thoracic surgery	22 patients; VA/VV-ECMO	Severe hemorrhage 13.6%	Blood cell destruction 4.5%; sepsis 13.6%	Successful decannulation 90.9%	Survival to discharge 77.2%	Survival pre- vs. postoperative ECMO p = 0.135; VV vs. VA p = 0.550
28	Kim et al. / 33536388	High-risk thoracic surgery with ECMO	63 patients	Not specifically quantified in abstract extraction	Not specified	Intraoperative arrest 17.5%	46 alive at 30 days	Arrest associated with mortality OR 24.44, 95% CI 1.82–327.60, p = 0.016; age OR 7.47, 95% CI 1.17–47.85, p = 0.034

29	Laverty et al. / 38079460	Tube thoracostomy in ARDS on VV-ECMO	25 COVID-19, 38 controls	Tube complications 89% vs. 33%	Not specified	Tube thoracostomy 36% vs. 24%	Not primary extracted outcome	Pneumothorax OR 2.8, 95% CI 0.95–7.9, p = 0.09; complications OR 16, 95% CI 1.6–201, p = 0.0498
30	Zhang et al. / 33908283	Complex thoracic surgery with ECMO	15 patients	Hemorrhage 26.7%	Venous thrombosis 26.7%	All patients weaned/extubated postoperatively	Hospital mortality 6.7%; 1-year survival 86%	Descriptive statistics only
31	Spaggiari et al. / 32814351	ECMO-assisted tracheal sleeve pneumonectomy	6 patients	One major postoperative complication: hemothorax	No cannula-positioning complications	Surgery feasible; all alive at follow-up	No perioperative mortality reported	Descriptive statistics only
32	McCann et al. / 31301640	Emergency laparotomy during ECMO	3.7% prevalence among 355 ECMO patients	Major hemorrhage uncommon	Emergency oxygenator change commonly required	Emergency laparotomy feasible	Survival to discharge 31%	Descriptive statistics only
33	Glowka et al. / 30096634	Decompressive laparotomy for ACS	11 DL among 175 ECMO patients	Not specifically quantified	Not specified	DL performed for ACS	Survival 27.3%; mortality 72.7%	Mortality DL vs. no DL p = 0.749; age p = 0.032; CCI >1 p = 0.004; SAPS II p = 0.013/p = 0.004
34	Schulz et	Emergency laparotomy	8 OA among	Abdominal	No enteroatmos	Median 7	Mortality 50%	Descriptive

	al. / 33102513	and open abdomen therapy	421 ECMO patients	packing for severe bleeding in all deceased patients	pheric fistula or abscess	surgical procedures/NPWT changes; revision in 3/8; fascial closure 75%		statistics only
35	Amata et al. / 32156186	Endoscopic treatment of GI bleeding during VV-ECMO	134 VV-ECMO; 14 GI bleeding	GI bleeding 10.4%; active bleeding in 11/14; complete endoscopic control in all active cases	Not specified	Endoscopic therapy successful in all active bleeding cases	No fatal GI bleeding; mortality not significantly different	ECMO duration longer with GI bleeding, p = 0.01; mortality p > 0.05
36	Park et al. / 41840082	VATS vs. clamshell bilateral LTx with ECMO	136 LTx; 31 VATS, 105 clamshell	Blood loss 832 vs. 2789 mL; fewer transfusions with VATS	Not dominant extracted outcome	Sternal wound complications and airway interventions only in clamshell group	Early postoperative outcomes favorable with VATS	Operative time p < 0.001; blood loss p < 0.001; FEV1/FVC at 1 month p < 0.001; 12-month FEV1 p = 0.05, FVC p = 0.04
37	Zubarevich et al. / 34674570	ECLS bridge to durable LVAD	35 patients	Re-sternotomy for bleeding in 42.9%.	Early LVAD thrombosis 2.9%; severe right heart failure 22.9%; AKI requiring dialysis 68.6%; respiratory	Durable LVAD implantation feasible	In-hospital 65.6%; 30-day 75.9%; 6-month 69.2%; 1-year 62.7% survival	Descriptive statistics only

					failure 77.1%.			
38	Potapov et al. / 33547707	LVAD implantation after VA-ECLS	531 patients; FS vs. LIS	More blood products with full sternotomy, 16 vs. 12 units; revision for bleeding 35.5% vs. 15.4%	Stroke 7.4% FS vs. 0% LIS	Less invasive surgery associated with less bleeding revision	30-day and 1-year survival similar	Blood products p = 0.033; revision for bleeding p = 0.016
39	Sorensen et al. / 32579786	Minimally invasive LVAD after ECMO/IABP bridge	83 patients; 11 ECMO	ECMO patients required more RBC transfusions	Stroke/infection/bleeding similar among groups	Outcomes comparable after stabilization	3- and 12-month survival ECMO 81% and 81%	Survival p = 0.45
40	Ljajikj et al. / 29049666	LVAD on ECLS with bivalirudin/HIT antibodies	21 bivalirudin vs. 36 heparin	Early re-exploration bleeding/tamponade 19% vs. 16.7%	Stroke, intracranial bleeding comparable	Re-thoracotomy >7 days comparable	Mortality up to 1 year comparable	OR 1.18, 95% CI 0.29–4.76, p = 0.820; matched p = 0.455
41	Patel et al. / 36466372	Robot-assisted CABG with peripheral ECMO	45 patients	Not specifically quantified	No stroke, MI, or access-vessel complications up to 30 days	One redo-CAB required sternotomy; 75.6% extubated within 6 h	30-day mortality 2.2%	Descriptive statistics only
42	Lutz et al. / 36975408	Thoracoabdominal aortic replacement and oncologic surgery	5 patients	Hematoma in 3; bleeding revision in 1	Bypass revision in 1; patency 100% during follow-up	All patients required surgical revision	No recurrent malignant disease during follow-up	Descriptive statistics only

43	Takabayashi et al. / 39497225	Critical PE requiring ECMO	76 patients	Major bleeding at 30 days 54.0%; procedure-site/surgery-related bleeding 22.4%	Not separately extracted	Treatment strategies included surgery, catheter intervention, thrombolysis, anticoagulation	30-day mortality 30.3%	Mortality: surgery 6.3%, catheter 43.8%, thrombolysis 25.0%, anticoagulation 39.3%
44	Johannesen et al. / 32142811	Intraoperative ECMO in thoracic surgery	3 patients	No ECMO-related complications observed	No ECMO complications observed	All decannulated after surgery	No procedure-related mortality reported	Descriptive statistics only
45	Boulos et al. / 31425255	Early decompressive laparotomy for intra-abdominal hypertension	9 VV-ECMO patients	No major surgical or bleeding complications reported.	Compromised return cannula flow emphasized as an indication for decompression.	Decompressive laparotomy improved ECMO flow, oxygenation, and pulmonary compliance.	Survival to discharge 56%.	Descriptive case-series data
46	Heward et al. / 29444601	Thoracic interventions in ECMO services	86 VV-ECMO patients; 25 underwent thoracic intervention	Thoracotomies, chest drains, bronchoscopies, blockers performed; bleeding not fully quantified	Not specifically reported	82 interventions; 11 thoracotomies; 49 chest drains; 13 rigid bronchoscopies; 3 re-explorations	Survival to discharge 72% among patients undergoing thoracic interventions	Descriptive statistics only
